# Bioactive Properties of Marine Phenolics

**DOI:** 10.3390/md18100501

**Published:** 2020-09-30

**Authors:** Raquel Mateos, José Ricardo Pérez-Correa, Herminia Domínguez

**Affiliations:** 1 Institute of Food Science, Technology and Nutrition (ICTAN-CSIC), Spanish National Research Council (CSIC), José Antonio Nováis 10, 28040 Madrid, Spain; raquel.mateos@ictan.csic.es; 2 Department of Chemical and Bioprocess Engineering, Pontificia Universidad Católica de Chile, Macul, Santiago 7810000, Chile; perez@ing.puc.cl; 3 CINBIO, Department of Chemical Engineering, Faculty of Sciences, Campus Ourense, Universidade de Vigo, As Lagoas, 32004 Ourense, Spain

**Keywords:** bromophenols, simple phenolics, flavonoids, phlorotannins, seawater, algae, seagrass, health benefits, biological activity

## Abstract

Phenolic compounds from marine organisms are far less studied than those from terrestrial sources since their structural diversity and variability require powerful analytical tools. However, both their biological relevance and potential properties make them an attractive group deserving increasing scientific interest. The use of efficient extraction and, in some cases, purification techniques can provide novel bioactives useful for food, nutraceutical, cosmeceutical and pharmaceutical applications. The bioactivity of marine phenolics is the consequence of their enzyme inhibitory effect and antimicrobial, antiviral, anticancer, antidiabetic, antioxidant, or anti-inflammatory activities. This review presents a survey of the major types of phenolic compounds found in marine sources, as well as their reputed effect in relation to the occurrence of dietary and lifestyle-related diseases, notably type 2 diabetes mellitus, obesity, metabolic syndrome, cancer and Alzheimer’s disease. In addition, the influence of marine phenolics on gut microbiota and other pathologies is also addressed.

## 1. Introduction

The occurrence of dietary and lifestyle-related diseases (type 2 diabetes mellitus, obesity, metabolic syndrome, cancer or neurodegenerative diseases) has become a health pandemic in developed countries. Global epidemiological studies have shown that countries where seaweeds are consumed on a regular basis have significantly fewer instances of obesity and dietary-related diseases [1]. Among marine metabolites with biological properties, phenolic compounds have attracted great interest. However, compared to those found in terrestrial sources, their study is recent and challenging in different aspects. Some families of phenolic compounds have been reported in both terrestrial and marine organisms but others, such as bromophenols and phlorotannins, are exclusively found in marine sources. The natural production of phenolic compounds in marine organisms has been associated with external factors, particularly with environmental stressing conditions, such as desiccation, salinity, UV radiation, nutrients availability, and temperature [2,3,4,5]. Variability and dependence with species, seasonality and environmental conditions occur for macroalgae [6,7,8] and seagrass [9,10,11] and with the growing conditions on microalgae [12].

Different extraction strategies have been successfully used, from conventional solvent extraction with water or with organic solvents to alternative techniques using either greener solvents or intensification tools to enhance yields and rates [13]. Enzymatic-assisted hydrolysis provided higher extraction rate and extraction yields, with lower time and cost, but appeared less effective for polyphenols because the extraction of other fractions such as proteins and saccharides was enhanced [14,15]. Ultrasonication aided in the disruption of marine algal biomass and the enhanced extraction of components [3,16,17,18]; hence, it can also be applied as a pretreatment [17]. However, degradation of bioactives could occur due to sonication induced effects such as high temperatures and radical’s generation. Cleaner and efficient polyphenol extraction processes using safer solvents are increasingly demanded. Supercritical CO_2_ extraction complies with these requirements and offers advantages derived from the tunablity of the solvation power by modifying pressure and temperature; however, due to its apolar character it requires the addition of polar modifiers. Most studies have been reported with crude solvent extracts; therefore, the properties cannot be ascribed to a single compound, and the synergistic effects among the components should be considered. Depending on the final use, a series of fractionation stages, would be required, because more active fractions can be obtained by purification of crude extracts [19,20]. 

The most basic phenolics quantification relies on the colorimetric Folin–Ciocalteu assay, but modern analytical tools have contributed to the provision of information on the complex structure of marine phenolics [21,22], usually with chromatographic, IR spectroscopic and NMR methods [9,10,23,24]. Advanced and coupled techniques such as HPLC–DAD–ESI/MS and UPLC–ESI–QTOF/MS analyses [5,7,20,25,26], LC–ESI–MS/MS [27], RRLC-ESI–MS [28], UPLC [29], UPLC–MS [25], UPLC–MS/MS TIC [4], ^1^D and ^2^D NMR techniques (^13^C-NMR, COSY, TOCSY, NOESY, HSQC) [24], are required to unveil the highly diverse and complex chemical structure of marine phenolics. The development of strategies for simultaneous determination and quantification of the different phenolic subclasses is needed [20]. Particularly interesting has been the identification of phlorotannins, which show an extremely large diversity and complexity, regarding the number or monomeric basic units, distribution of hydroxyl groups and structural conformations of isomers [7,25,29]. In addition, their combination with preconcentration, and hydrolysis allowed simultaneous determination of phenolics in minutes [28]. Biological resources including seaweed may contain toxic compounds, such as heavy metals, and the evaluation of toxicity is required prior to focusing on any other activity [30,31].

Abundant reviews of the bioactive properties of marine phenolics can be found [32,33,34,35,36]. Most of them have been focused on seaweeds, but other marine organisms deserve interest as potential worldwide distributed and ubiquitous sources of phenolic compounds. Furthermore, the extensive variety of biological activities with potential to improve human and animal health, as well as the possibility of using these compounds for the formulation of novel products, configurates the food and feed applications as an efficient route of administration to maintain health and for preventing and treating different diseases. This review presents an overview of the major phenolic compounds found in marine sources and discusses their relevant biological properties in relation to lifestyle related diseases.

## 2. Marine Phenolics: Sources and Phenolic Composition

### 2.1. Families of Phenolic Compounds Identified in Marine Sources

**Figure 1:**
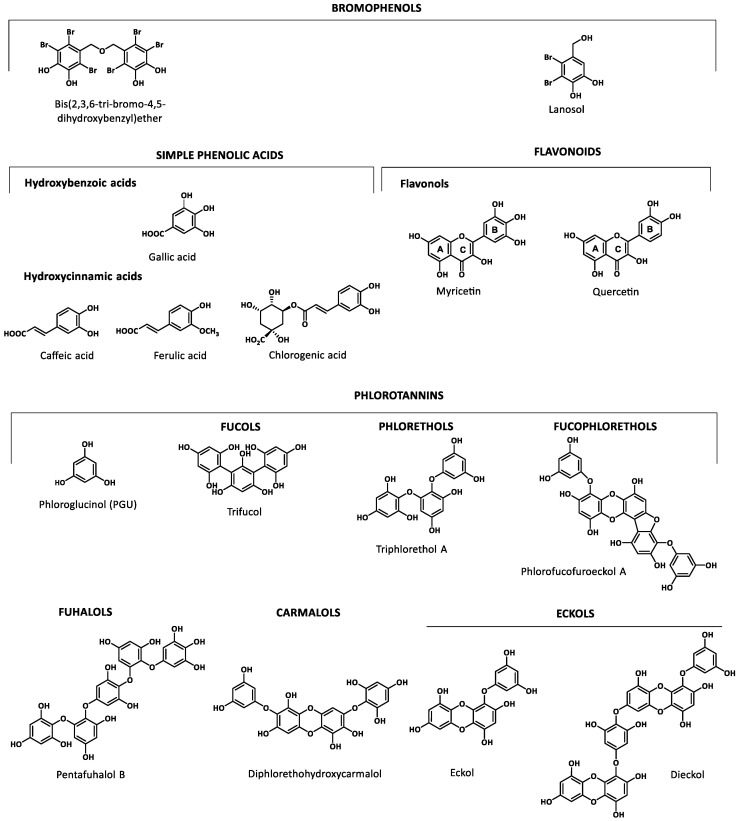
Examples of the families of phenolic compounds found in marine sources: bromophenols, simple phenolic acids and flavonoids, different types of phlorotannins (fucols, phlorethols, fucophlorethols, fuhalols, carmalols and eckols, as well as phloroglucinol monomeric unit).

Marine organisms are a rich source of phenolics that include bromophenolic compounds, simple phenolic acids and flavonoids as well as phlorotannins. Figure 1 shows the basic structure of some key classes of the marine phenolics identified. Examples of each class were selected based on their biological relevance in the reported studies. 

Bromophenolic compounds have been found in several macroalgae (red, green and brown) and cyanobacteria. They can be transferred through the food chain from macroalgae to invertebrate grazers to fish. Since some of them have toxic properties similar to those of anthropogenic contaminants, their characterization is needed [37]. The lack of reports regarding the industrial production of commercially available bromophenols (hydroxylated and methoxylated bromodiphenyl ethers) suggest that they should come from natural sources and from biotransformation of natural and anthropogenic compounds [38]. Red algae are the major source of natural marine bromophenols [39], but other organisms such as fish, shrimps and crabs ingest them through the food chain. Cade et al. [38] found polybrominated diphenyl ethers (PBDEs) at higher concentration in finfish than in shellfish. Among shellfish, bivalves (clams and mussels) tended to have higher levels of hydroxylated and methoxylated PBDEs than other types of seafood. Koch and Sures [40] have compiled information on the concentrations of tribromophenols in aquatic organisms, ranging from 7 to 1600 ng/g algal ww, 0.3 to 2360 ng/g crustacean ww, 0.9 to 198 ng/g mollusks dw, 3.7 to 230 ng/g fish ww.

Phenolic acids and flavonoids have also been found in marine sources. Among phenolic acids, there are two major groups, hydroxycinnamic acids and hydroxybenzoic acids, whereas flavonols, belonging to flavonoids is the most abundant group of compounds identified in marine organisms [20,41,42]. Phlorotannins, exclusively found in brown seaweeds, are complex polymers of phloroglucinol (1,3,5-trihydroxybenzene). This structurally heterogeneous group presents a complex chemical composition, diverse linkage positions and a degree of polymerization (126 Da–650 kDa) [21] which determine its biological properties. The structural classification is based on the inter-monomeric linkages: fucols possess only aryl–aryl linkages, phlorethols aryl–ether linkages, fuhalols possess only ether linkages and additional OH groups in every third ring, fucophlorethols possess aryl–aryl and aryl–ether units, carmalols are derived from phlorethols and possess a dibenzodioxin moiety, and eckols that possess at least one three-ring moiety with a dibenzodioxin moiety substituted by a phenoxyl group at C-4 [43,44,45].

### 2.2. Sources

#### 2.2.1. Seawater

The most abundant phenolic compounds found in seawater are sinapic acid, catechin, myricetin, kaempherol and protocatechuic acid (found at 0.8–2.8 nM/L), whereas vanillic acid, coumaric acid, ferulic acid, and rutin are below 0.5 nM/L [46]. In a recent study on the presence of free phenolic compounds in Antarctic sea water, Zangrando et al. [42] concluded that the release from phytoplankton could be the origin of phenolics in seawater, since diatoms produce exudates that contain phenolic compounds. Other possible but less plausible sources could be the intrusion of circumpolar deep water that may transport oceanic lignin; the melting of glaciers, which contain lignin that can be degraded in the snow; photooxidation in water; the photochemical and microbiological degradation of lignin contained in dissolved organic material. These authors have found vanillin, vanillic acid, acetovanillone and p-coumaric acid, both in the dissolved and particulate fractions in seawater samples, with syringic acid, syringaldehyde and homovanillic acid at residual concentrations. Bidleman et al. [37] also reported the presence of the bromophenol lanosol (2,3-dibromo-4,5-dihydroxybenzyl alcohol) in seawater.

#### 2.2.2. Microalgae

Microalgae conform a highly ecologically diverse group of unicellular eukaryotic organisms; they are the most important primary source of biomass in aquatic ecosystems. They are able to produce a wide variety of commercially interesting compounds, such as lipids, carbohydrates, phenolics, carotenoids, sterols, vitamins, and other bioactives [47]. Microalgae offer advantages over terrestrial sources derived from their metabolic diversity and adaptive flexibility, the efficient photosynthesis and high growth rate, the possibility of large scale cultivation, simple nutritional requirements, and their ability to accumulate or secrete metabolites [48]. Microalgae can grow in different habitats such as fresh water, saltwater and marine environments. They can even grow on industrial wastewaters [49]. The valuable bioactives with pharmaceutical, food, feed, and cosmetic applications [50,51] from microalgae could be relevant regarding the higher profitability of the cultivation processes and could complement the energetic application [47]. In fact, the extraction of phenolic compounds from microalgae biomass does not interfere with already established processes such as biofuel production [27].

Microalgae produce protective antioxidant compounds in response to stress damage caused by UV radiation, temperature variation, excessive light, and others. In some cases, these are not influencing factors. Gómez et al. [52] observed that the accumulation of phenolic compounds in some microalgae was independent of the illumination condition. The production of flavonoids and polyphenols could be favored with the adequate control of selected variables of the culture process [12]. Non-natural factors, such as CuO nanoparticles, can induce the production of phenolics in *Nannochloropsis oculata* [53], lowering growth rates as well as chlorophyll and carotenoids content. Moreover, CuO nanoparticles damaged the membrane as well as increased the activity of antioxidant endogenous enzymes, such as catalase, ascorbate peroxidase, polyphenol oxidase and lactate dehydrogenase.

Some phenolics in marine microorganisms are released into the environment to form metal complexes in order to acquire micronutrients or to sequester toxic metals, and their presence can stimulate the growth of diatoms. Catechin, sinapic acid, apigenin, quercitrin, kaempferol, epicatechin, gentisic acid, syringic acid, chlorogenic acid, vanillic acid, ferulic acid, caffeic acid, protocatechuic acid, coumaric acid, rutin and gallic acid have been reported in the exudates from diatoms [42,54,55].

Data in Table 1, Table 2 and Table 3 summarize the phenolic compounds reported in marine organisms and their in vitro antioxidant characteristics, which could be used as a preliminary indication of potential bioactivities. Phenolic compounds can be efficient antioxidants acting with different mechanisms, as scavengers of singlet oxygen and free radicals, reducing agents, chelating agents, inhibiting specific oxidative enzymes or can act by mixed mechanisms. Assays to determine the reducing and antiradical properties against 2,2-diphenyl-1-picrylhydracil (DPPH), as well as 2,2′-azino-bis (3-ethylbenzothiazoline-6-sulphonic acid) (ABTS), superoxide and hydroxyl radicals, are usually preferred to screen the most active extracts from natural sources. Data in Table 1 confirm that phenolic acids, and particularly hydroxycinnamic acids, are the major families identified in microalgae.

Considering the diversity of phenolic compounds found in marine organisms, and the influence of composition on the activity, the selection of the extraction solvent is important and should be chosen with care, either individually or in mixtures [3,17]. Some examples are cited to illustrate this fact. In a comparative study, acetone provided the highest phenolic content in extracts from *Isochrysis galbana*, *Tetraselmis* sp. and *Scenedesmus* sp. The highest radical scavenging activity was observed in the acetone extract of *I. galbana*, the maximum Fe (II) chelating capacity in the hexane extract of *Scenedesmus* sp. and the in vitro inhibition of acetylcholinesterase in the water and ether extracts of both microalgae. Whereas the antiradical properties of the polar extracts can be ascribed to phenolics, in the non-polar extracts the activity could be due to fatty acids or to other lipophilic components [56]. Aqueous and methanolic extracts provided higher phenolic yield and reducing power from *Nannochloropsis gaditana* than acetone, dichloromethane or hexane; however, acetone provided the highest DPPH radical scavenging activity and cytotoxicity against human lung cancer cells (A549) [57]. Moreover, the methanolic extracts of *Chaetoceros curvisetus*, *Thalassiosira subtilis* and *Odontella aurita* were more active than those in acetone and in hexane [58].

In some cases, a linear relationship between phenolic content and antioxidant and biological properties has been established. Phenolic content is correlated with DPPH radical scavenging activity [18,59] and also with antitumoral properties [56]. Solvent extracts from *Nannochloropsis oceanica* showed reducing and antiradical properties and those from *Skeletonema costatum* and *Chroococcus turgidus* showed chelating ability; both properties are correlated with the phenolic content [59]. However, this correlation was found to be insignificant in other extracts, suggesting that these might not be major contributors to the antioxidant capacities [60]. Safafar et al. [49] reported that phenolic compounds were the major contributors to the antioxidant activity in microalgal extracts, but also carotenoids contributed to the DPPH radical scavenging activity, ferrous reduction power (FRAP), and ABTS-radical scavenging capacity activity. Maadame et al. [3] did not find correlation between the antioxidant capacities and the phenolic and carotenoids content in ethanolic extracts [57]. The low phenolic content (0.3–20 mg GAE/g DW) in microalgal extracts [48,56,58] could suggest that other compounds could be responsible for the observed activities, such as carotenoids, fatty acids, sterols, vitamins as well as other compounds such as micosporine-like aminoacids (MAAs) [61]. The TEAC (Trolox equivalent antioxidant capacity) values and antiproliferative activities of phytoplankton extracts show a strong positive correlation with the amount of the total carotenoids and micosporine-like aminoacids, but were negatively correlated with the amounts of phenolic compounds [18]. 

**Table 1:** Phenolic compounds identified in different marine organisms: microalgae, cyanobacteria, fungus, seagrasses and sponges.

Marine Organism Extraction Chemical Analysis	Phenolic Compounds Antioxidant Activity (When Provided)	Ref.
**Marine-derived Fungus**		
*Alternaria* sp. SCSIO41014 from sponge AC, EtOAc, US HPLC-UV, HRESIMS, NMR, ECD, XRay	Perylenequinone derivatives; altenusin derivative; phenol derivatives	[62]
*Arthrinium* sp. MeOH, MeCN HPLC, HRESIMS, NMR	2-(2,3-Dihydroxy-5-methyl benzoyl)-6-hydroxybenzoic acid	[63]
*Aspergillus sydowii*, from the sponge *Stelletta* sp. EtOAc, BuOH HPLC, UV, IR, HRESIMS, NMR, OP	Diorcinolic acid; β-d-glucopyranosyl aspergillusene A; diphenylethers; chromone; xanthone first glycoside of phenolic bisabolane sesquiterpenes	[64]
*Aspergillus* sp. from the sponge *Xestospongia testudinaria* EtOAc, AC RP-HPLC, HRESIMS, NMR	Phenolic bisabolane sesquiterpenoid dimers (disydonols A–C), (S)-(+)-sydonol	[65]
*Aspergillus* sp. from the sponge *Chondrilla nucula* EtOAc HPLC–PDA, UV, HRESIMS, NMR, OP	Phenolic bisabolane sesquiterpenes; asperchondol A; asperchondol B	[66]
*Aspergillus* sp., from the sponge *Chondrilla nucula* EtOAc, BuOH HPLC, UV, IR, HRESIMS, NMR, OP	Phenolic bisabolane sesquiterpenes; asperchondols A and B; diphenyl ethers	[64]
*Aspergillus versicolor*, deep-sea fungus EtOAc, BuOH HPLC, IR, TLC, HRESIMS, NMR, OP, ECD	Aspergilols A–F; diorcinal; cordyol E; 4-carboxydiorcinal; 4-methoxycarbonyldiorcinol; 4-carbethoxydiorcinal; cordyol C; methylgerfelin; violaceol II; averythrin; averantin, 1’-*O*-methylaverantin; lecanoric acid; orsellic acid; orcinol; 1- methylpyrogallol; fumaric acid ABTS = 0.1–5.4 mmol Trolox/g	[67]
*Cladosporium cladosporioides* from *Sargassum wightii* EtOAc LC-MS	N-(2-Iodophenyl)-2-[2-oxo-5-(thiophen-2-yl)-2,3-dihydro-1,3,4-oxadiazol-3-yl] acetamide; 2-[3-chloro-4-(4-chlorophenoxy)phenyl]-1,3-dioxo-2,3-dihydro-1H-isoindole-5-carboxylic acid; 2-(2,4-dichlorophenyl)-2-oxoethyl 3,4-dihydro-2H-1,5-benzodioxepine-7-carboxylate; 4-bromo-N*′*-(4-fluoro-1-benzothiophene-2-carbonyl)-1H-pyrrole-2-carbohydrazide; (1*R*,2*R*,5*S*)-2-[3-({2-[(2,4-dichlorophenyl)methyl]-2H-1,2,3,4-tetrazol-5-yl}methyl)-4-methyl-5 sulfanylidene-4,5-dihydro-1H-1,2,4-triazol-1-yl]-6,8-dioxabicyclo [3.2.1]octan-4-one; methyl 2-({[5-bromo-2-(4 methoxybenzamido)phenyl] (phenyl)methyl}amino) acetate; 2-[4-(2,4-dichlorophenoxy)phenyl]-5-phenyl-octahydro-1H-isoindole-1,3-dione; N-({2-[(3,4-dichlorophenyl) methoxy]naphthalen-1-yl}methyl)-2,3-dihydro-1,4-benzodioxin- 6-amine; 2-({[(2,4-dichlorophenyl) carbamoyl] methyl}(propyl)amino)-N-(2,2,2-trifluoroethyl) acetamide; N-(4-bromo-2-fluorophenyl)-6-(2-tert-butylhydrazin1-yl)-5-nitropyrimidin-4-amine; N-(2-{3-[(3,4-di chlorophenyl)methyl]-2-oxo-1,3-diazina EC_50,DPPH_ = 50 mg/mL; EC_50,DPPH, AS_ = 18 mg/mL; RP = 0.81 mg/g	[68]
*Penicillium brevicompactum* EtOAc RP-HPLC, UV, HRESIMS, NMR	Anthranilic acid; syringic acid; sinapic acid; acetosyringone IC_50, DPPH_ = 20–30 µg/mL	[69]
*Penicillium janthinellum* EtOAc, MeOH HPLC, UV, IR, HRESIMS, GC-MS, NMR	6-(2-Acetyl-3,5-dihydroxybenzyl)-4-hydroxy-3-methyl-2H-pyran-2-one; 7-hydroxy-2-(hydroxymethyl)-5-methyl-4H-chromen4-one; 3,5-dihydroxy-2-(2-(2-hydroxy-6-methylphenyl)-2-oxoethyl)-4-methylbenzaldehyde; 3-hydroxy-5-methylphenyl 2,4-dihydroxy-6-methylbenzoate; lecanoric acid; orsellinic acid; orcinol	[70]
*Penicillium griseofulvum* EtOAc, BuOH HPLC, UV, IR, HRESIMS, NMR, OP	4,6-Dimethylcurvulinic acid	[64]
*Penicillium expansum* 091006 from the mangrove plant *Excoecaria agallocha* EtOAc, AC HPLC, UV, IR, HRESIMS, TLC, NMR, OP, ECD	Phenolic bisabolane sesquiterpenoid and diphenyl ether units, expansols A and B, (*S*)-(+)-11-dehydrosydonic acid,(7*S*,11*S*)-(+)-12-acetoxysydonic acid, (*S*)-(+)-sydonic acid, diorcinol,(*S*)-(+)-2-[3-hydroxy-4-(2-methoxy-6-methylheptan-2-yl)benzyl]-5- (3-hydroxy-5-methylphenoxy)-3-methylphenol, *S*-(+)-2-[3-hydroxy-4-(2-hydroxy-6- methylheptan-2-yl)benzyl]-5-(3-hydroxy-5-methylphenoxy)-3-methylphenol, (*S*)-(+)-3-hydroxy-4-(2-hydroxy-6-methylhept-6-en-2-yl)benzoic acid, and 4-[(2*S*,6*S*)-7-acetoxy-2-hydroxy-6-methylheptan-2-yl]-3-hydroxybenzoic acid	[71]
*Scopulariopsis* sp. EtOAc, MeOH, W, H HPLC-PDA, RP-HPLC, LC-MS, HRESIMS, TLC, NMR, OP	12-Dimethoxypinselin; 12-*O*-acetyl-AGI-B4, 11,12-dihydroxysydonic acid; 1-hydroxyboivinianic acid	[72]
ZSDS1-F11 from the sponge *Phakellia fusca* EtOAc, AC TLC, CC, HRESIMS, NMR	Phenolic bisabolane sesquiterpenoid and diphenyl ether units; expansols A–F; diorcinol	[73]
**Cyanobacteria**		
*Anabaena* C5 E, US HPLC-MS/MS	Quinic acid; catechin EC_50 DPPH_ = 0.1 mg/mL; FRAP = 11.4 mg ASE/g	[74]
*Arthrospira* S1, S2 EtOH, US HPLC-MS/MS	Catechin EC_50, DPPH_ = 0.1 mg/mL; FRAP = 15.1–21.0 mg ASE/g	[74]
*Calothrix* sp. SI-SV MeOH, W HPLC-UV/VIS	Rutin; tannic acid; orcinol; phloroglucinol; protocatechuic acid EC_50, ABTS_ = 65.79 μg/mL, EC_50, DPPH_ = 69.38 μg/mL	[75]
*Leptolyngbya* sp. MeOH, W HPLC-UV/VIS	Rutin; tannic acid; orcinol; phloroglucinol; protocatechuic acid SI-SM (EC_50, ABTS_ = 63.45, EC_50, DPPH_ = 67.49 μg/mL)	[75]
*Nostoc commune* MeOH, W RP-HPLC-DAD	Gallic and chlorogenic acids	[76]
*Nostoc* sp EtOH, US HPLC-MS/MS	Gallic acid; chlorogenic acid; quinic acid; catechin; epicatechin; kaempferol; rutin; apiin IC_50, DPPH_ = 0.04–9.47 mg/mL; FRAP = 8.4–13.7 mg ASE/g	[74]
**Microalgae**		
*Ankistrodesmus* sp. MeOH, W RP-HPLC-DAD	Protocatechuic acid DPPH scavenging_10 mg/mL_ = 29%	[76]
*Euglena cantabrica* MeOH, W RP-HPLC-DAD	Gallic acid; protocatechuic acid; chlorogenic acid; (+) catechin; (−)epicatechin DPPH scavenging_10 mg/mL_ = 71%	[76]
*Nannochloropsis* sp. EtOH, MeOH, H RP-HPLC-UV HPLC–ESI–MS/MS	Phenolic acids: chlorogenic; caffeic; gallic; protocatechuic; hydroxybenzoic; syringic; vanillic; ferulic	[27]
*Spirulina* sp. RP-HPLC-UV HPLC-ESI-MS/MS	Protocatechuic; gallic; chlorogenic; vanillic; hydroxybenzoic; syringic; vanillic acids	[27]
*Spirogyra* sp. MeOH, W RP-HPLC-DAD	Gallic acid DPPH scavenging_10 mg/mL_ = 62%	[76]
**Seagrasses**		
*Cymodocea nodosa* MeOH, CH_2_CL_2_ HPLC-DAD, LC/MS-ESI, NMR	Diosmetin 7-sulfate; caftaric acid; coutaric acid	[77]
*Halodule wrightii*, *Thalassia testudinum* Agar, W HPLC	p-hydroxybenzoic acid; ferulic acid; p-coumaric acid; syringic acid; gallic acid	[78]
*Halophila stipulacea* MeOH, EtOAc, Hexane HR-LC-MS/MS GNPS	Luteolin; apigenin; matairesinol; cirsimarin; spiraeoside; 2,4-dihydroxyheptadec-16-ynyl acetate; 3-hydroxy-4-methoxycinnamic acid, alpha-cyano-4-hydroxycinnamic	[79]
*Posidonia oceanica* (L.) EtOH, W, Formic acid HPLC-ESI-MS/MS	Procyanidin B2; procyanidin C2; isorhamnetin-3-*O*-glucoside; quercetin-3-*O*-glucoside; quercetin-3-*O*-malonylglucoside; isorhamnetin-3-*O*-malonylglucoside EC_50, DPPH_ = 32 µg/mL	[80]
*Ruppia cirrhosa* (Petagna) Grande, *Ruppia maritima* L. MeOH, W, EtOAc HPLC-DAD, HR-LCMS-ESI+/TOF, NMR	Chicoric acid; quercetin 3-*O*-β-d-(6’’-*O*-malonyl)-glucopyranoside; quercetin 3-*O*-β-d-galactopyranoside; quercetin 3-*O*-β-d-glucopyranoside; quercetin 3-*O*-β-d-(6′’-*O*-malonyl)galactopyranoside; isorhamnetin 3-*O*-β-d-galactopyranoside; isorhamnetin 3-*O*-β-d-glucopyranoside; isorhamnetin 3-*O*-β-d-(6´´-*O*-malonyl) galactopyranoside; isorhamnetin 3-*O*-β-d-(6′’-*O*-malonyl)-glucopyranoside EC_50 DPPH_ = 23–176 µg/mL	[81]
*Syringodium isoetifolium* MeOH HPLC-EI-MS	Caftaric acid; 2 3-(4-Hydroxyphenyl)lactic acid; caffeic acid; caffeoyl-4′-*O*-phenyllactate; 3-phenyllactic acid; 4-coumaric acid; chicoric acid DPPH = 5.4 mg TE/g; ABTS = 9.6 mg TE; CUPRAC = 18.7 mg TE/g; FRAP = 9.5 mgTE/g; Chelating ability = 9.17 mg EDTAE/g	[82]
*Thalassia testudinum* AC, W, AA	3,4-Dihydroxybenzoic acid; p-hydroxybenzoic acid; p-coumaric acid; vanillin	[83]
*T. testudinum* EtOH, W RP-HPLC LC-MS, NMR	3,4-Dihydroxybenzoic acid, p-hydroxybenzoic acid, p-coumaric acid and vanillin	[84]
*Zostera asiatica* and *Z. marina* HPLC-MS	Rosmarinic acid; luteolin; 7,3′-disulfate luteolin ROS scavenger; protecting or enhancing endogenous antioxidants; metal chelation	[85]
*Z. marina* Hexane, AC HPLC-MS, NMR	Deoxycymodienol; isotedarene A	[86]
*Z. marina* SPA, IPA HPLC-MS/MS	3-Hydroxyhexanoic acid; 4-hydroxynonenoic acid; p-coumaric acid; caffeic acid; ferulic acid; zosteric acid; apigenin; luteolin; diosmetin; apigenin-7-sulfate; rosmarinic acid; luteolin-7-sulfate; diosmetin-7-sulfate; kaempferol-7,4′-dimethylether-3-*O*-sulfate	[5]
*Z. noltii* MeOH HPLC, NMR	Rosmarinic acid; caffeic acid; zosteric acid	[87]
*Z. noltei* MeOH HPLC-PDA-MS-ESI-QTOF, NMR	Rosmarinic acid; apigenin-7-*O*-glucoside; luteolin; apigenin; diosmetin; acacetin; luteolin-7-sulfate; apigenin-7-sulfate; diosmetin-7-sulfate; acacetin-7-sulfate	[88]
*Zostera noltei* leaves MeOH, W HPLC-DAD, LC-MS, NMR	Apigenin 7-sulfate; diosmetin 7-sulfate	[89]
*Zostera noltii*, *Z. marina*	Apigenin 7-sulphate; luteolin 7-sulphate; diosmetin 7-sulphate; rosmarinic acid; luteolin 7-glucoside; apigenin 7-glucoside; apigenin; luteolin 7-(6′’-malonyl) glucoside; apigenin 7-(6′’-malonyl) glucoside	[81]
*Zostera muelleri* MeOH, AA RP-HPLC	Proanthocyanidins; gallic acid; rosmarinic acid	[90]
**Sponges**		
*Didiscus aceratus* MeOH, CH_2_Cl_2_, H HRESIMS, NMR	(S)-(+)-Curcuphenol; 10β-hydroxycurcudiol; 10α-hydroxycurcudiol; dicurcuphenols A–E; dicurcuphenol ether F	[91]
*Hyrtios erectus* MeOH, EtOAc HRAPCIMS, HRESIMS, NMR	Phenolic alkenes; erectuseneols A−F	[92]
*Myrmekioderma* sp. MeOH, CH_2_Cl_2_, EtOAc, BuOH, hexane HRESIMS, NMR	1-(2,4-Dihydroxy-5-methylphenyl)ethan-1-one; (1′Z)-2-(1′,5′-dimethylhexa-1′,4′-dieny1)-5- methylbenzene-1,4-diol; 1,8-epoxy-1(6),2,4,7,10-bisaborapentaen-4-ol; 6-(3-hydroxy-6-methyl-1,5- heptadien-2-yl)-3-methylbenzene-1,4-diol; 4-hydroxy-3,7-dimethyl-7-(3-methylbut-2- en-1-yl)benzofuran-15-one; 6-(2-methoxy-6-methylhept-5-en-2-yl)-3-methylbenzene-1,4-diol; 9-(3,3-dimethyloxiran-2-yl)-1,7-dimethyl-7-chromen-4-ol	[93]
*Myrmekioderma* sp. MeOH, EtOAc HRAPCIMS, HRESIMS, NMR	(R)-Biscurcudiol; (S)-biscurcudiol; myrmekiodermaral; myrmekioperoxide A; myrmekioperoxide B (4); myrmekiodermaral; (+)-curcudiol; (+)-dehydrocurcudiol; abolene; abolene epimer at C-5′; (+)-oxoabolene; (+)-curcuphenol; 5′α-hydroxycurcudiol; 5′β- hydroxycurcudiol; curcuepoxide A; curcuepoxide B	[94]

AA: ascorbic acid; AC: acetone; ASE: ascorbic acid equivalents; BuOH: butanol; CA: chelating ability; CAA: antioxidant assay for cellular antioxidant activity [95]; CLPAA: cellular lipid peroxidation antioxidant activity assay [95]; EA: ethyl acetate; ECD: electronic circular dichroism; EtOAc: ethyl acetate; EtOH: ethanol; GNPS: global natural product social molecular networking; H: hexane; HRAPCIMS: high resolution atmospheric pressure chemical ionization mass spectrometry; HRESIMS: high resolution electrospray ionization mass spectrometry; IPA: isopropanol; LPIA: lipid peroxidation inhibition assay [96]; MeCN: acetonitrile; MeOH: methanol; OP: polarimetry; PDA: photodiode array; PGU: phloroglucinol units; RP: reducing power; RP-HPLC: reversed phase HPLC; SPA: solid phase adsorption; SRSA: superoxide radical scavenging assay [96]; TAA: total antioxidant capacity [97]; TLC: thin-layer chromatography; US: ultrasound; W: water.

#### 2.2.3. Macroalgae

##### Bromophenols

Among the halogenated secondary metabolites synthesized by seaweeds, brominated ones are more usual due to the availability of chloride and bromide ions in seawater; iodine and fluorine are less frequent. Whereas iodination can be found in brown algae, bromine or chlorine metabolites are more abundant in red and in green seaweeds [98]. The most abundant bromophenolic compounds found in macroalgae are bromophenols and their transformation products bromoanisoles, hydroxylated and methoxylated bromodiphenyl ethers and polybrominated dibenzo-p-dioxins [2,37]. Other brominated compounds have also been identified in macroalgae, such as brominated sesquiterpenes [99]. 

Some bromophenols identified in seaweeds are shown in Table 2 and Table 3. Specifically, 2,4,6-tribromophenol is widely distributed, coming from environmental contaminants, pesticides and from marine organisms, which produce it as a defense against predators and biofouling. Machado et al. [100] found bromoform (1.7 mg/g), dibromochloromethane (15.8 μg/g), bromochloroacetic acid (9.8 μg/g) and dibromoacetic acid (0.9 μg/g) in *Asparagopsis taxiformis*, and Bidleman et al. [37] found bromoanisols at more than 1000 pg/g in *Ascophyllum nodosum*, *Ceramium tenuicorne*, *Ceramium virgatum*, *Fucus radicans*, *Fucus serratus*, *Fucus vesiculosus*, *Saccharina latissima*, *Laminaria digitata*, and *Acrosiphonia/Spongomorpha sp*. The presence of these compounds can be associated with off-flavors. Among the bromophenols identified in prawn species, probably obtained from marine algae and bryozoan from the diet, are 2-and 4-bromophenol, 2,4-dibromophenol, 2,4,6-tribromophenol and 2,6-dibromophenol. The latter confers an iodoform off-flavor at 60 ng/kg and it was found in prawn, reaching more than 200 μg/kg in the eastern king prawn. This off-flavor can be reduced by handling and processing [101]. Kim et al. [102] reported that 3-bromo-4,5-dihydroxybenzaldehyde exerted antioxidant effects in skin cells subjected to oxidative stress, by increasing the protein and mRNA levels of glutathione synthesizing enzymes, enhancing the production of reduced glutathione in HaCaT cells and protecting cells against oxidative stress via the activation of the NF-E2-related factor.

Their extraction can be achieved with organic solvents, i.e., methanol or methanol-dichloromethane [37], but yields can vary with other factors. Seasonal variations and different profiles among species, locations and environmental conditions have been observed [40], their production being induced by environmentally stressing conditions, such as the presence of herbivores and the elevated levels of light and salinity [2].

##### Simple Phenolics

The presence of benzoic and cinnamic acids has been reported, particularly in brown seaweeds, which also present flavonoids [20,103]. Brown seaweeds present higher contents of benzoic and cinnamic acids (1 mg/g) than red (0.2 3 mg/g) and green (0.01–0.9 mg/g) seaweeds [26,104,105]. Higher values (1–9 mg/g) have been reported for gallic acid in green and red seaweeds [106]. These authors reported catechin content up to 14 mg/g in red seaweeds and up to 11.5 mg/g in green ones, whereas in brown seaweeds reached up to 11 mg/g. Phloroglucinol derivatives are the major phenolics in brown seaweeds, and flavonoids account for 35% of the total, the most abundant being gallic, chlorogenic acid, caffeic acid, ferulic acid [20].

The phenolic levels correlated positively with elevated irradiance exposure and temperature and their content differs among different parts of the seaweed. Extracts of the thallus were more active than extracts of the receptacles, and the solvent was also important, the best being acetone, ethanol, and water. The drying stage should also be optimized, since degradation may occur, i.e., dried material provided lower yield and less active extracts than frozen ones [24].

**Table 2:** Phenolic compounds found in brown seaweeds.

Seaweed Extraction Chemical Analysis	Compounds Antioxidant Activity (When Provided)	Ref.
*A. nodosum*, *F. spiralis* MeOH, AC, Hexane UPLC, MS, NMR	Phlorotannins (4–6, 9–12 PGU)	[107]
*A. nodosum*, *Fucus spiralis*, *F. vesiculosus*, *Pelvetia canaliculata*, *Saccharina longicruris* MeOH, W UPLC, HRMS	Phlorotannins (3–50 PGU)	[108]
*Carpophyllum flexuosum*, *Carpophyllum plumosum*, *Ecklonia radiata* W, MAE HPLC-DAD-ESI-MS, NMR	Bifuhalol, bifuhalol dimer, bifuhalol trimer, hydroxytrifuhalol, trifuhalol, tetrafuhalol DPPH = 2.7–37.4 mg GAE/g; FRAP = 4.4–62.1 mg GAE/g	[109]
*Cystoseira barbata* TFA, W LC-QTOF-MS	Phloroglucinol, rutin, phlorofucofuroeckol, 3-*O*-rutinosyl-kaempferol, catechin-catechin-*O*-gallate, gallocatechin, gallocatechin-*O*-glucuronide, 1-hydroxy-2-(β-d-glucopyranosyloxy)-9,10 anthraquinone, 2-*O*-(6,9,12-octadecatrienoyl)-3-*O*-(nonadecanoyl)glyceryl β-galactopyranoside, chlorogenic acid butyl ester, phloroglucinol, quercetin EC_50, DPPH_ = 11.7 µg/mL; EC_50, OH_ = 11.4 µg/mL; EC_50, RP_ = 51 g/mL; EC_50, CA_ = 40 g/mL	[110]
*C. barbata* AC, MeOH, W UHPLC-DAD-QTOF-MS	Fucophlorethol and eckol derivatives (3–7 PGU) EC_50, DPPH_ = 14 µg/mL; EC_50, ABTS_ = 0.5 µM Trolox; EC_50, RP_ = 16–35 µg/mL	[111]
*Cystoseira nodicaulis*, *Cystoseira tamariscifolia*, *Cystoseira usneoides*, *F. spiralis* AC, Hexane, W HPLC-DAD-ESI-MS^n^	Fucophloroethol, fucodiphloroethol, fucotriphloroethol, 7-phloroeckol, phlorofucofuroeckol, bieckol, dieckol EC_50, SRSA_ = 0.93–4.02 mg/mL; EC_50, LPIA_ = 2.32–>9.1 mg/mL	[96]
*Cystoseira nodicaulis*, *F. serratus*, *F. vesiculosus*, *Himanthalia elongata* EtOH, W UPLC-ESI-MS	Phlorotannin (3–16 PGU) EC_50, DPPH_ = 4–28 μg/mL; FRAP = 101–307 μg TE/mg	[21]
*Durvillaea antarctica*, *Lessonia spicata* EtOH, EE, EtOAc, W HPLC-MS-MRM	Phlorotannin (3–8 PGU), flavonoids EC_50, DPPH_ = 0.97–1.24 mg/mL; FRAP = 2.95–6.20 mM TE/kg; ORAC = 4.75–25.9 μM TE/g	[112]
*Eisenia bicyclis* EtOH HPLC-PDA	Eckol, phlorofucofuroeckol-A, dieckol, 6,6′-bieckol, 8,8′-bieckol	[113]
*Ecklonia cava* EtOH UPLC-PDA	Phloroglucinol, eckol, eckstolonol, triphlorethol-A, dieckol	[114]
*E. cava* EtOH, US HPLC-DAD-ESI/MS, NMR	Dieckol, phlorofucofuroeckol-A, 2,7-phloroglucinol-6,6-bieckol, pyrogallol-phloroglucinol-6,6-bieckol	[115]
*E. cava* EtOH, W RP-HPLC	Dieckol ABTS = 1.3 g VCE/g; DPPH = 0.4 g VCE/g	[116]
*Ecklonia stolonifera* EtOH, W HPLC-PDA, NMR	2-phloroeckol, dioxinodehydroeckol, eckol, phlorofucofuroeckol B, 6,6’-bieckol, dieckol, 974-B, phlorofucofuroeckol A	[117]
*F. vesiculosus* MeOH, W Q-ToF-MS, UPLC-TQD-MS/MS-MRM	Phlorotannins (3–18 PGU) EC_50, DPPH_ = 18.2 μg/mL	[118]
*F. vesiculosus* AC, EtOAc, EtOH, MeOH, W HPLC-DAD-ESI/MS*^n^*	Fucodiphlorethol A, trifucodiphlorethol isomers, phlorotannins (3–10 PGU) EC_50, DPPH_ = 2.79–4.23 μg/mL; Fe^2+^-CA = 25.1–47.6%; RP = 17.8–910.7 mg ASEs/g	[119]
*F. vesiculosus* AC, W UPLC-DAD-ESI/MS*^n^*	Fucols, fucophlorethols, fuhalols, phlorotannin derivatives (3–22 PGU), fucofurodiphlorethol, fucofurotriphlorethol, fucofuropentaphlorethol	[120]
*Halidrys siliquosa* AC, W MALDI-TOF-MS, NMR	Diphlorethol, triphlorethol, trifuhalol, tetrafuhalol EC_50, DPPH_ = 0.02–1.00 mg/mL; EC_50, RP_ = 0.06–0.62 mg/mL; EC_50, NBT_ = 0.66–2.44 mg/mL; ORAC = 5.39 μmol TE/mg; BCB = 0.21–1.50 g/mL	[121]
*H. elongata* MeOH, W HPLC-DAD, HPLC-ESI-MS/MS	Phloroglucinol, gallic acid, chlorogenic acid, caffeic acid, ferulic acid, hydroxybenzaldehyde, kaempferol, myricetin, quercetin EC_50, DPPH_ = 14.5 μg/mL	[20]
*Hydroclathrus clathratus*, *Padina minor*, *Padina* sp., *Sargassum oligosystum*, *Sargassum aff. bataanens*, *Sargassum* sp. MeOH, W GC-MS-EI-SIM	2,4,6-tribromophenol; 2,4,6-tribromoanisol; 2′-hydroxy-2,3′,4,5′- tetrabromodiphenyl ether; 2’-methoxy-2,3′,4,5′-tetrabromodiphenyl ether; 6-hydroxy-2,2′,4,4′-tetrabromodiphenyl ether; 6-methoxy-2,2′,4,4′- tetrabromodiphenyl ether; 2′,6-dihydroxy-2,3′,4,5′-tetrabomodiphenyl ether; 2′,6-dimethoxy-2,3′,4,5′-tetrabromodiphenyl ether; 2,2′-dihydroxy- 3,3′,5,5′-tetrabromodiphenyl; 2,2′-dimethoxy-3,3′,5,5′-tetrabromodiphenyl	[122]
*L. digitata* MeOH, W RP-UPLC-UV-MS*^n^*, MALDI-TOF-MS, NMR	Di–fuhalols (6–7 PGU), fucols (3–7 PGU), fucophlorethols (3–16 PGU), fuhalols (4–5 PGU), phlorethols (3–18 PGU)	[123]
*Leathesia nana* CH_2_Cl_2_, EtOH, W HRESIMS, NMR	2,2′,3,3′-tetrabromo-4,4′,5,5′-tetrahydroxydiphenylmethane; 3-bromo-4- (2,3-dibromo-4,5-dihydroxybenzyl)-5-methoxymethylpyrocatechol; 2,3,3′-tribromo-4,4′,5,5′-tetrahydroxyl-1′-ethyloxymethyldiphenyl methane; 2,3-dibromo-4,5-dihydroxybenzaldehyde; 2,3-dibromo-4,5- dihydroxybenzyl alcohol; 2,3-dibromo-4,5-dihydroxybenzyl methyl ether; 2,3-dibromo- 4,5-dihydroxybenzyl ethyl ether; 3,5-dibromo-4-hydroxybenzaldehyde; 3,5-dibromo-4-hydroxybenzoic acid; 3-bromo-4,5-dihydroxybenzoic acid methyl ester, 3-bromo-5-hydroxy-4-methoxybenzoic acid, 3-bromo-4-hydroxybenzoic acid EC_50, DPPH_ = 14.5 µg/mL	[124]
*Lessonia trabeculate* MeOH, W, MWE HPLC-DAD-ESI-MS/MS	Phlorotannins derivatives (2–3 PGU), gallocatechin derivative, p-coumaric acid derivative	[103]
*Padina tetrastromatica* PE, MeOH, CHCl_3_, MEK, Soxhlet HPLC-UV, UPLC-MS/MS	Fucophlorethol (2–18 PGU) EC_50, DPPH_ = 17 µg/mL	[125]
*Sargassum fusiforme* EtOH, W UPLC-DAD-ESI-MS/MS	Eckol, dieckol, dioxinodehydroeckol, fuhalols (2–12 PGU), phlorethols/fucols/fucophlorethols (2–11 PGU), eckols (2–8 PGU) EC_50, DPPH_ = 15–150 µg/mL; FRAP = 1.29 mg TE/mg	[126]
*Sargassum muticum* EtOH, W, PHLE LC × LC-DAD-ESI-MS/MS	Decafuhalol, dihydroxytetrafuhalol, dihydroxypentafuhalol, dihydroxyhexafuhalol, dihydroxyheptafuhalol, dihydroxyoctafuhalol, dihydroxynonafuhalol, heptaphlorethol, hexafuhalol, hexaphlorethol, hydroxytetrafuhalol, hydroxypentafuhalol, hydroxyhexafuhalol, nonafuhalol, octafuhalol, pentafuhalol, pentaphlorethol, tetrafuhalol, trifuhalol, trihydroxyhexafuhalol, trihydroxyheptafuhalol, trihydroxyoctafuhalol ABTS = 0.65–2.29 mmol TE/g	[127]
*Silvetia compressa* EtOH, US HPLC-DAD, HPLC-TOF-MS	Dihydroxytetrafuhalol, dieckol, eckol derivative, eckstolonol, 7-phloroeckol (3 PGU), dihydroxypentafuhalol, phlorofucofuroeckol A, pentafuhalol, trifuhalol	[128]

ABTS: 2,2’-azino-bis (3-ethylbenzothiazoline-6-sulphonic acid); AC: acetone; ASE: ascorbic acid equivalents; BCBM: β-carotene bleaching; CA: chelating ability; DPPH: 2,2-diphenyl-1-picrylhydrazyl; EE: ethyl ether; EI-SIM: electron ionization selected ion monitoring mode; EtOAc: ethyl acetate; EtOH: ethanol; FRAP: ferric reducing antioxidant power; LC × LC: two-dimensional liquid chromatography; LPIA: Lipid peroxidation inhibition assay; MALDI-TOF: matrix-assisted laser desorption/ionization time-of-flight; MEK: methyl ethyl ketone; MeOH: methanol; MRM: multiple reaction monitoring; MWE: microwave-assisted extraction; NBT: superoxide anion scavenging test; ORAC: oxygen radical absorbance capacity; PDA: photodiode array; PGU: phloroglucinol units; PHLE: pressurized hot liquid extraction; RP: reducing power; RP-UPLC: reversed phase UPLC; SRSA: superoxide radical scavenging assay [96]; TE: trolox equivalents; TEAC: trolox equivalent antioxidant capacity; TFA: trifluoroacetic acid; TQD-MS: tandem quadrupole mass spectrometer; US: ultrasound; W: water.

##### Phlorotannins

These are structural components of the cell wall in brown algae, and also play a function in macroalgal chemical defense comparable to those of secondary metabolites, such as protection from UV radiation and defense against grazing [4]. The species and cultivation conditions affect the composition. Lopes et al. [7] found five and six ringed phloroglucinol oligomers in wild grown and aquaculture-grown *F. vesiculosus*, trimers and tetramers in extracts from *Fucus guiryi*, *F. serratus* and *F. spiralis*. Fucophlorethols are dominant in *Fucus sp*, exhibiting molecular weights ranging from 370 to 746 Da, and relatively low degree of polymerization (3–6 phloroglucinol units, PGU). Moreover, isomers of fucophlorethol, dioxinodehydroeckol, difucophlorethol, fucodiphlorethol, bisfucophlorethol, fucofuroeckol, trifucophlorethol, fucotriphlorethol, tetrafucophlorethol, and fucotetraphlorethol were identified.

Heffernan et al. [21] reported that most low molecular weight (LMW) phlorotannins presented 4–16 monomers of phloroglucinol. The level of isomerization differed among macroalgal species and *F. vesiculosus* showed up to 61 compounds with 12 PGU. Species-specific phenolic profiles, with varying degrees of composition, polymerization and isomerization have been described and the antiradical activity observed was not only due to higher phlorotannin concentrations but to their geometric arrangement and the position of the free hydroxyl groups [4]. *F. vesiculosus* low molecular weight fractions were predominantly composed of phlorotannins between 498 and 994 Da (4–8 PGUs), in *P. canaliculata* most structures presented 9–14 PGUs, whereas in *H. elongata* most phlorotannins were composed of 8–13 PGU.

The influence of the solvent has been reported in a number of studies either as extractant or as fractionation medium, i.e., Murugan and Iyer [129] found higher ferrous ion chelation and growth inhibition of MG-63 cells by methanolic and aqueous extracts from *Caulerpa peltata*, *Gelidiella acerosa*, *Padina gymnospora*, and *S. wightii*. However, the higher extraction of phenols and flavonoids was found with chloroform and ethyl acetate, as well as the DPPH radical scavenging and growth inhibitory activities in cancer cells. Aravindan et al. [130] selected dichloromethane and ethyl acetate fractions from *Dictyota dichotoma*, *Hormophysa triquerta*, *Spatoglossum asperum*, *Stoechospermum marginatum* and *P. tetrastromatica* for their high levels of phenolics, antioxidants and inhibitors of pancreatic tumorigenic cells (MiaPaCa-2, Panc-1, BXPC-3 and Panc-3.27) growth. The use of intensification techniques can enhance the extraction yields. Kadam et al. [16] reported that under ultrasound-assisted extraction of *A. nodosum* in acidic media, the extraction of high molecular weight phenolic compounds was facilitated. However, other bioactives were also solubilized in a short time and crude solvent extracts contained several non-phenolic components, such as carbohydrates, amino acids and pigments. Further purification strategies have been tried, i.e., a multistep scheme with successive precipitation of lipophilic compounds and further chromatographic fractionation [24], solvent partition and membrane fractionation [21], solvent partition and column chromatography [19,22], adsorption, washing and further elution [131], or chromatography and then membrane processing by ultrafiltration and dialysis [132].

**Table 3:** Phenolic compounds found in red and green seaweeds.

Seaweed Extraction Chemical Analysis	Compounds Antioxidant Activity (When Provided)	Ref.
**Green seaweeds**		
*Caulerpa lentillifera*, *C. taxifolia*, *Chaetomorpha crassa*, *Chara* sp., *Chlorodesmis* sp., *Cladophora* sp. MeOH, W GC-MS-EI-SIM	2,4,6-Tribromophenol; 2,4,6-tribromoanisol; 2′-hydroxy-2,3′,4,5′-tetrabromodiphenyl ether; 2’-methoxy-2,3′,4,5′-tetrabromodiphenyl ether; 6-hydroxy-2,2′,4,4′-tetrabromodiphenyl ether; 6-methoxy-2,2′,4,4′-tetrabromodiphenyl ether; 2′,6-dihydroxy-2,3′,4,5′-tetrabromodiphenyl ether; 2′,6-dimethoxy-2,3′,4,5′-tetrabromodiphenyl ether; 2,2′-dihydroxy-3,3′,5,5′-tetrabromodiphenyl; 2,2′-dimethoxy-3,3′,5,5′-tetrabromodiphenyl	[122]
*Dasycladus vermicularis* MeOH UPLC-MS/MS	4-(Sulfooxy)phenylacetic acid; 4-(sulfooxy)benzoic acid	[133]
**Red seaweeds**		
*Acanthophora specifera*, *Ceratodictyon spongiosum*, *Gracilaria edulis*, *Hydropuntia edulis*, *Halymenia* sp., *Jania adhaeren*, *Jania* sp., *Kappaphycus alvarezii* MeOH, W GC-MS-EI-SIM	2,4,6-Tribromophenol; 2,4,6-tribromoanisol; 2′-hydroxy-2,3′,4,5′-tetrabromodiphenyl ether; 2’-methoxy-2,3′,4,5′-tetrabromodiphenyl ether; 6-hydroxy-2,2′,4,4′-tetrabromodiphenyl ether; 6-methoxy-2,2′,4,4′-tetrabromodiphenyl ether; 2′,6-dihydroxy-2,3′,4,5′-tetrabromodiphenyl ether; 2′,6-dimethoxy-2,3′,4,5′-tetrabromodiphenyl ether; 2,2′-dihydroxy-3,3′,5,5′-tetrabromodiphenyl; 2,2′-dimethoxy-3,3′,5,5′-tetrabomodiphenyl	[122]
*Asparagopsis taxiformis* W, MeOH, CH_2_Cl_2_, H GC-MS	Bromoform, dibromochloromethane, bromochloroacetic acid, dibromoacetic acid	[100]
*Bostrychia radicans* MeOH, H, EtOAc GC-MS, NMR	N,4-dihydroxy-N-(2′-hydroxyethyl)-benzamide; N,4-dihydroxy-N-(2′-hydroxyethyl)-benzeneacetamide; methyl 4-hydroxymandelate; methyl 2-hydroxy-3-(4-hydroxyphenyl)-propanoate	[134]
*C. tenuicorne* H + DEt + 2-P GC-MS, ECNI	Phenols, hydroxylated, and methoxylated penta- and hexabrominated diphenyl ethers	[135]
*C. tenuicorne* H + DEt + 2-P GC-MS, ECNI	Hydroxylated polybrominated diphenyl ethers 2′-hydroxy-2,3′,4,5′-tetrabromodiphenyl ether; 6-hydroxy-2,2′,4,4′-tetrabromodiphenyl ether	[136]
*Laurencia nipponica*, *Odonthalia corymbifera*, *Polysiphonia morrowii* A, W, MeOH LC-MS, NMR	3,5-Dibromo-4-hydroxybenzaldehyde; 3-bromo-4,5-dihydroxybenzyl ether; 3-bromo-4,5-dihydroxybenzyl alcohol; 5-((2,3-dibromo-4,5-dihydroxybenzyloxy)methyl)-3,4-dibromobenzene-1,2-diol; 5-(2-bromo-3,4-dihydroxy-6-(hydroxymethyl) benzyl)-3,4-dibromobenzene-1,2-diol	[137]
*O. corymbifera*, *Neorhodomela aculeata*, *Symphyocladia latiuscula* A, W, MeOH LC-MS, NMR	n-Butyl 2,3-dibromo-4,5-dihydroxybenzyl ether; 3-bromo-4-(2,3-dibromo-4,5-dihydroxybenzyl)-5-methoxymethylpyrocatechol; 2,3-dibromo-4,5- dihydroxybenzyl alcohol; 2,3-dibromo-4,5-dihydroxybenzyl methyl ether; bis-(2,3,6-tribromo-4,5-dihydroxybenzyl) ether; 2,3,6-tribromo-4,5-dihydroxybenzyl methyl ether; 2,2′,3,3′-tetrabromo-4,4′,5,5′-tetrahydroxydiphenylmethane; 5-(2-bromo-3,4-dihydroxy-6-(hydroxymethyl)benzyl)-3,4-dibromobenzene- 1,2-diol; 5-((2,3-dibromo-4,5-dihydroxybenzyloxy)methyl)-3,4-dibromobenzene-1,2-diol	[138]
*Odonthalia corymbifera* MeOH, EtOAc NMR	Odonthalol, odonthadione	[139]
*Polysiphonia decipiens* 3:1 MeOH:CH_2_Cl_2_ NMR	α-*O*-Methyllanosol; lanosol; 5-(2-bromo-3,4-dihydroxy-6-(hydroxymethyl)benzyl)-3,4-dibromobenzene-1, 2-diol; 5-(2-bromo-3, 4-dihydroxy-6-(methoxymethyl)benzyl)-3, 4-dibromobenzene-1, 2-diol; rhodomelol; polysiphonol	[140]
*Polysiphonia morrowii* W, MeOH, CH_2_Cl_2_ NMR	3-bromo-4,5-dihydroxybenzyl methyl ether; 3-bromo-4,5-dihydroxybenzaldehyde	[141]
*P. morrowii* W, MeOH ESI-MS, NMR	bis (3-Bromo-4,5-dihydroxybenzyl) ether	[142]
*Rhodomela confervoides* EtOH NMR	3-(2,3-Dibromo-4,5-dihydroxybenzyl) pyrrolidine-2,5-dione; methyl 4-(2,3-dibromo-4,5-dihydroxybenzylamino)-4-oxobutanoate; 4-(2,3-dibromo-4,5-dihydroxybenzylamino)-4-oxobutanoic acid; 3-bromo-5-hydroxy-4-methoxybenzamide; 2-(3-bromo-5-hydroxy-4-methoxyphenyl)acetamide; 3-bromo-4,5-bis(2,3-dibromo-4,5-dihydroxybenzyl) pyrocatechol; methyl 1-(2-(2,3-dibromo-4,5-dihydroxybenzyl)-3-bromo-4,5- dihydroxybenzyl)-5-oxopyrrolidine-2-carboxylate; 5-((2,3-dibromo-4,5-dihydroxybenzyloxy)methyl)-3,4-dibromobenzene-1,2-diol; 5-(2-bromo-3,4-dihydroxy-6-(hydroxymethyl)benzyl)-3,4-dibromobenzene-1,2-diol; 5-(2-bromo-3,4-dihydroxy-6-(methoxymethyl) benzyl)-3,4-dibromobenzene-1,2-diol; 5-(2-bromo-6-(ethoxymethyl)-3,4-dihydroxybenzyl)-3,4-dibromobenzene-1,2-diol; 5-(2,3-dibromo-4,5-dihydroxybenzyl)- 3,4-dibromobenzene-1,2-diol; 1-(2,3-dibromo-4,5-dihydroxybenzyl)-5-oxopyrrolidine-2-carboxylic acid; methyl 1-(2,3-dibromo-4,5-dihydroxybenzyl)-5-oxopyrrolidine-2-carboxylate EC_50, DPPH_ = 5.2–23.6 µmol/L; ABTS = 2.1–3.6 mmol TE/L	[143]
*Symphyocladia latiuscula* EtOH NMR	2,3-Dibromo-4,5-dihydroxybenzyl methyl ether, 3,5-dibromo-4-hydroxybenzoic acid; 2,3,6-tribromo-4,5-dihydroxymethylbenzene; 2,3,6-tribromo-4,5-dihydroxybenzaldehyde; 2,3,6-tribromo-4,5-dihydroxybenzyl methyl ether; bis(2,3,6-tribromo-4,5-dihydroxyphenyl)methane; 1,2-bis(2,3,6-tribromo-4,5-dihydroxyphenyl)-ethane; 1-(2,3,6-tribromo-4,5-dihydroxybenzyl)-pyrrolidin-2-one	[144]
*S. latiuscula* EtOH, EtOAc, W HRMS, NMR, MS	1-[2,5-dibromo-3,4-dihydroxy-6-(2,3,6-tribromo-4,5-dihydroxybenzyl)benzyl]pyrrolidin-2-one; methyl 4-{(2,3,6-tribromo-4,5-dihydroxybenzyl)[(2,3,6-tribromo-4,5-dihydroxybenzyl)carbamoyl] amino}butanoate; methyl 4-{(2,5-dibromo-3,4-dihydroxybenzyl)[(2,3,6-tribromo-4,5-dihydroxybenzyl)carbamoyl]amino} butanoate; 2,5-dibromo-3,4-dihydroxy-6-(2,3,6-tribromo-4,5-dihydroxybenzyl)benzyl methyl ether EC_50, DPPH_ = 14.5, 20.5 µg/L	[145,146]
*S. latiuscula* EtOH, EtOAc, W ESIMS, NMR	Bromocatechol conjugates (symphyocladins)	[147,148]
*S. latiuscula* MeOH, CH_2_Cl_2_, EtOAc, BuOH, W ESIMS, NMR	2,3,6-tribromo-4,5-dihydroxybenzyl alcohol; 2,3,6-tribromo-4,5-dihydroxybenzyl methyl ether; bis-(2,3,6-tribromo-4,5-dihydroxybenzyl) ether	[149]
*Vertebrata lanosa* MeOH, EtOAc ESIMS, NMR	2,3-Dibromo-4,5-dihydroxybenzylaldehyde; 2,2′,3-tribromo-3′,4,4′,5-tetrahydroxy-6′-hydroxymethyldiphenylmethane; bis(2, 3-dibromo-4,5-dihydroxylbenzyl) ether; 5,5″-oxybis(methylene)bis (3-bromo-4-(2′,3′-dibromo-4′,5′-dihydroxylbenzyl)benzene-1,2-diol) ORAC = 0.08–0.33 μg TE/mL; CAA_inhib. 10 μg/mL_ = 68%; CLPAA_inhib. 10 μg/mL_ = 100%	[95]
*V. lanosa*	Methylrhodomelol; lanosol; lanosol methyl ether; 2-amino-5-(3-(2,3-dibromo-4,5-dihydroxybenzyl)ureido)pentanoic acid; 3-bromo-4-(2,3-dibromo-4,5-dihydroxybenzyl)-5-methoxymethylpyrocatechol; 5-((2,3-dibromo-4,5-dihydroxybenzyloxy)methyl)-3,4-dibromobenzene-1,2-diol; 2,2′,3,3′-tetrabromo-4,4′,5,5′-tetrahydroxydiphenylmethane	[150]

ABTS: 2, 2′-azino-bis (3-ethylbenzothiazoline-6-sulphonic acid); ASE: ascorbic acid equivalents CA: chelating ability; BCBM: β-carotene bleaching; CAA: antioxidant assay for cellular antioxidant activity [95]; CLPAA: cellular lipid peroxidation antioxidant activity assay [95]; DCM: dichloromethane; DEtE: diethylether; DPPH: 2,2-diphenyl-1-picrylhydrazyl; ECNI: electron capture negative ionization; EtOAc: ethyl acetate; EtOH: ethanol; FRAP: ferric reducing antioxidant power; H: hexane; LPIA: lipid peroxidation inhibition assay; MeOH: methanol; NBT: superoxide anion scavenging test; ORAC: oxygen radical absorbance capacity; 2-P: 2-propanol; PGU: phloroglucinol units; RP: reducing power; SRSA: superoxide radical scavenging assay [96]; TE: trolox equivalents; TEAC: trolox equivalent antioxidant capacity; W: water.

Kirke et al. [118] reported that low molecular weight phlorotannin fractions (<3 kDa) from *F. vesiculosus* in powder form remained stable under storage for 10 weeks, when exposed to temperature and oxygen. Although, when suspended in an aqueous matrix, this fraction underwent oxidation when exposed to atmospheric oxygen and 50 °C, and both the DPPH radical scavenging activity and the content of phlorotannins with 6–16 PGUs decreased.

Since other compounds found in the crude seaweed extracts could be responsible for the biological activities, the correlation between phenolic content and antioxidant properties was not always found in seaweed extracts. These relationships should also consider the type of activity assayed since some of them share the same mechanisms [151]. Furthermore, not only the phenolic content is determinant, but also their structure. In brown seaweeds, the classical correlation of phenolic content and radical scavenging has been established with the antiradical properties and molecular weight. Phlorotannin-enriched fractions from water and aqueous ethanolic extracts of *A. nodosum* and *Pelvetia canaliculata* contain predominantly larger phlorotannins (DP 6–13) compared to *F. spiralis* (DP 4–6) [25]. The 3.5–100 kDa and/or >100 kDa fractions from the cold water and aqueous ethanolic extracts showed higher phenolic content, radical scavenging abilities and ferric reducing antioxidant power (FRAP) than the <3.5 kDa, which could enhance their activity after a reverse-phase flash chromatography fractionation [152]. In a study on *F. vesiculosus*, Bogolitsyn et al. [22] concluded that the highest radical scavenging activity was observed for average molecular weights from 8 to 18 kDa and the activity decreased with increasing molecular weight from 18 to 49 kDa. This effect has been ascribed to the formation of intramolecular and intermolecular hydrogen bonds between hydroxyl groups, causing conformational changes in phlorotannin molecules and, therefore, mutual shielding and a decrease in the availability of active centers. *Ascophylllum nodosum* purified oligophenolic fraction was more active than the crude fraction as ABTS scavengers, and the fraction containing phenolic compounds with a MW ≥50 kDa was the most active and showed higher correlation with the content of phenolic compounds [132]. Whereas the radical scavenging activity and reducing power showed correlation, particularly in brown seaweeds, the chelating properties did not, and were higher in green seaweed extracts, because the major activity could come from the saccharidic fraction [13]. The FRAP activity displayed a stronger correlation with the phlorotannin content than the radical scavenging capacity, as well as the phenolic content, molecular weight and structural arrangement [4]. However, other authors did not find any significant correlation between the total phenolic content of the extracts and the inhibition of red blood cell hemolysis and lipid peroxidation [153], or the antioxidant activity (DPPH and β-carotene bleaching assays) [154].

#### 2.2.4. Seagrasses

Compared to algae, seagrasses are scarcely exploited [11,33]. The worldwide distributed *Zostera* genus produces large amounts of leaf material. This is not utilized, representing an abundant waste which could be proposed to recover valuable compounds and therefore compensate the costs of cleaning beaches and shorelines used for recreational purposes [10].

Seagrasses are a rich source of (poly)phenolics, including simple and sulfated phenolic acids, such as zosteric acid, and condensed tannins [33,83]. Rosmarinic acid and caffeic acid (0.4–19.2 mg/g) were the major phenolic components in leaves and roots-rhizomes of eelgrass (*Zostera marina* L.), and higher concentrations have been found during spring in the younger leaves and roots-rhizomes [9,78]. Rosmarinic acid was also reported as an active phenolic in the methanolic extracts from detritus of *Z. noltii* and Z. *marina* (2.2–18.0 and 1.3–11.2 mg/g, respectively) [10]. Extraction yields, seasonally dependent for the two species, vary in the range of 9.3–19.7% (g/g dw) for *Z. noltii*, and 9.6–31% for *Z. marina*; near 85% of the rosmarinic acid was recovered from the crude methanolic extract using ethyl acetate [10]. Chicoric acid was the major compound in *Ruppia* sp, with 30 mg/g; twice the total flavonoid content [81]. High concentrations of phenolics in methanolic *Zostera* extracts correspond to higher growth inhibition of the toxic red tide dinoflagellate *A. catenella* [23]. In sc-CO_2_ extracts with ethanol or methanol cosolvents, the phenolic content and radical scavenging capacity correlated well with the cytotoxicity on tumoral cell lines; this high activity might be due to the high content of phenylpropanoids [155]. The supercritical CO_2_ extraction of phenolic compounds from *Zostera marina* residues using 20% ethanol as co-solvent enhanced the solubilization of polar compounds (chicoric, p-coumaric, rosmarinic, benzoic, ferulic and caffeic) [29,155], reaching phenolic contents comparable to those found in the ethanolic and methanolic Soxhlet extracts; the DPPH radical-scavenging activities were also similar.

#### 2.2.5. Sponges

Despite being a rich source of highly bioactive compounds [93], there are few studies in the literature regarding the extraction and identification of polyphenols in sponges. Methanol and dichloromethane were normally used for extraction, while new phenolic compounds have been identified using HRAPCIMS, HRESIMS and NMR. Bisabolenes are particularly interesting polyphenolic compounds found in sponges. These phenolics are characterized by a C-7 absolute stereochemistry. All sponge bisabolenes possess a unique 7S configuration, while other marine and terrestrial bisabolenes possess a 7R configuration [91]. (S)-(+)-curcuphenol, a member of this family commonly found in sponges, presents several biological activities [91].

## 3. Bioactive Properties of Marine Phenolics

Epidemiological, clinical and nutritional studies strongly support the evidence that dietary polyphenols play important roles in human health. Their regular consumption has been associated with a reduced risk of different chronic diseases, including cardiovascular diseases (CVDs), cancer and neurodegenerative disorders [156]. Marine polyphenols have also attracted much attention because, similar to other polyphenols, they are bioactive compounds with potential health benefits in numerous human diseases due to their enzyme inhibitory effect and antimicrobial, antiviral, anticancer, antidiabetic, antioxidant, or anti-inflammatory activities; however, most of the findings are based on in vitro assays and animal testing on rodents.

Studies demonstrating the multi-targeted protective effect of marine phenolics, focused on the most prevalent diseases such as type 2 diabetes mellitus, obesity, metabolic syndrome, Alzheimer’s disease and cancer, are included in this section. In addition, the influence of marine phenolics on gut human microbiota and other infectious have been also addressed (Table 4, Table 5, Table 6, Table 7, Table 8, Table 9 and Table 10). 

### 3.1. Type 2 Diabetes Mellitus

Type 2 diabetes mellitus (T2DM) is one of the most common non-communicable diseases in the world, which can be attributed to hyperglycemia characterized by a high glucose concentration circulating in the blood, and has a marked impact on the quality of life [157]. This disease leads to higher risk of premature death and is associated with several health problems such as vision loss, kidney failure, leg amputation, nerve damage, heart attack and stroke [158]. Due to its chronic nature, T2DM is also associated with several comorbidities such as metabolic syndrome (MetS), overweight and obesity, hypertension, non-alcoholic hepatic steatosis, coronary disease, and neuropathy, among others [159].

Phlorotannins of edible seaweeds are involved in various antidiabetic mechanisms: inhibition of starch-digesting enzymes α-amylase and α-glucosidase, protein tyrosine phosphatase 1B (PTP1B) enzyme inhibition, modulation of glucose-induced oxidative stress and reduction in glucose levels and lipid peroxidation, among others [160,161]. There are a few recent reviews that summarize the huge number of in vitro studies along with minor number of in vivo studies focused in evaluating the antidiabetic activity of polyphenols [35,36,160,162] or bioactive components of seaweeds [161,163]. Key in vitro studies along with the recent in vivo studies about antidiabetic activity of marine polyphenols are detailed below (Table 4).

Alpha-amylase, located in the pancreas, and α-glucosidase, at the brush border of intestinal cells, are two key enzymes involved in carbohydrate metabolism [164]. These enzymes break down carbohydrates into monosaccharides that are absorbed into the bloodstream, resulting in a rise in blood glucose following a meal. Oral glucosidase inhibitor drugs are the common clinical treatment for T2DM; however, long-term use can result in side effects such as renal tumors and hepatic injury [164]. Hence, looking for alternative natural products with no side effects is an active research area. Most brown seaweeds belonging to the genus *Ecklonia* and family *Lessoniaceae* have been reported to exhibit antidiabetic activities [160]. Five isolated phlorotannins from *E. cava*, fucodiphloroethol G, dieckol, 6,6′-bieckol, 7-phloroeckol, phlorofucofuroeckol-A, have shown a marked α-glucosidase inhibition with 19.5 μM, 10.8 μM, 22.2 μM, 49.5 μM and 19.7 μM, respectively, as well as some α-amylase inhibitory effect with IC_50_ values of >500 μM, 125 μM, >500 μM, 250 μM and >500 μM, respectively [165]. Phlorotannins extracted from *A. nodosum* [166,167], *Alaria marginata* and *Fucus distichus* [168] are also able to inhibit α-amylase and α-glucosidase, while those of *F. vesiculosus* [160] and *L. trabeculate* [103] inhibit α-glucosidase activity, and *L. trabeculate* [103] inhibits lipase activity (Table 4). Generally, seaweed extracts and isolated compounds exhibited more inhibitory potency towards α-glucosidase compared to α-amylase (see IC_50_ values in Table 4), which is desirable since high inhibition of α-amylase activity has been suggested to cause abnormal fermentation of undigested carbohydrates by the colonic microbiota [169]. This promising inhibitory activity towards the enzymes involved in the digestion of carbohydrates has led to the development of polyphenol-rich extracts from seaweeds as alternative drugs to treat T2DM. Catarino et al. [120] obtained crude extracts and semi-purified phlorotannins from *F. vesiculosus* containing fucols, fucophlorethols, fuhalols and several other phlorotannin derivatives, tentatively identified as fucofurodiphlorethol, fucofurotriphlorethol and fucofuropentaphlorethol. These extracts showed the potential to control the activities of α-amylase, pancreatic lipase, and particularly α-glucosidase, for which a greater inhibitory effect was observed compared to the pharmaceutical drug acarbose (IC_50_~4.5 – 0.82 μg/mL against 206 μg/mL, respectively). Park et al. [170] isolated minor phlorotannin derivatives from *E. cava* that effectively inhibited the activity of α-glucosidase, with IC_50_ values ranging from 2.3 to 59.8 μM; they obtained the kinetic parameters of the receptor–ligand binding by a fluorescence-quenching study. In the same line, Lopes et al. [8] isolated phlorotannins from four edible *Fucus* species (*F. guiryi*, *F. serratus*, *F. spiralis* and *F. vesiculosus*). These were chemically characterized using mass spectrometry-based techniques (HPLC–DAD–ESI/MS and UPLC–ESI–QTOF/MS). The isolated phlorotannins showed inhibitory activity against α-amylase and α-glucosidase, being particularly important in the activity of the latter, with IC_50_ values significantly lower (between 2.48 and 4.77 μg/mL) than those obtained for the pharmacological inhibitors acarbose and miglitol (between 56.43 and 1835.37 μg/mL). *F. guiryi* and *F. serratus* were the most active of the tested *Fucus* species. In addition, xanthine oxidase activity, an enzymatic system usually overexpressed in diabetes and responsible for producing deleterious free radicals, was also inhibited, related with the antioxidant activity associated to phlorotannins [8]. 

Protein tyrosine phosphatase 1B (PTP1B) is a major negative regulator of insulin signaling and is localized on the cytoplasmic surface of the endoplasmic reticulum in hepatic, muscular and adipose tissues. Due to its ubiquity in the insulin-targeted tissues and its role in insulin resistance development [142], inhibition of PTP1B activity would be a target for the treatment of T2DM and obesity. Ezzat et al. [171] reviewed the in vitro studies focused on evaluating the inhibitory activity of PTP1B marine polyphenols. Xu et al. [172] studied the inhibitory activity of a marine-derived bromophenol compound (3,4-dibromo-5-(2-bromo-3,4-dihydroxy-6-(ethoxymethyl)benzyl)benzene- 1,2-diol) isolated from the red alga *Rhodomela confervoides* in insulin-resistant C2C12 myotubes. This bromophenol has the ability to inhibit PTP1B activity (IC_50_ 0.84 μM), permeate into cells and bind to the catalytic domain of PTP1B in vitro, activate insulin signaling and potentiate insulin sensitivity in C2C12 myotubes as well as enhance glucose uptake. Similarly, 3-bromo-4,5-bis(2,3-dibromo-4,5-dihydroxybenzyl)-1,2-benzenediol isolated from the red alga *Rhodomela confervoides* was able to activate insulin signaling and prevent palmitate-induced insulin resistance by intrinsic PTP1B inhibition (IC_50_ 2.0 μM). Moreover, this compound also activated the fatty acid oxidation signaling in palmitate-exposed C2C12 myotubes [173].

Glycated insulin is commonly found in T2DM patients and is less effective in controlling glucose homeostasis and stimulating glucose uptake than non-glycated insulin [174]. Non-enzymatic protein glycation is an irreversible modification between reducing sugars and primary amino groups and leads to the production of advanced glycation end-products (AGEs) [175], whose accumulation causes various diabetic complications such as nephropathy, retinopathy and atherosclerosis as well as stimulates the development of neurodegenerative diseases such as Alzheimer’s disease (AD) [176]. The inhibition of AGEs formation is another approach being explored in managing hyperglycemia using seaweeds. Crude phlorotannins contained in the Japanese *Lessoniaceae* exhibited an inhibitory effect on the formation of fluorescence bound AGEs (IC_50_ 0.43–0.53 mg/mL), and among the purified phlorotannins (phlorofucofuroeckol-A, eckol, phloroglucinol, fucofuroeckol A, dieckol and 8,8′-bieckol), phlorofucofuroeckol A showed the highest inhibitory activity (IC_50_ 4.1–4.8 × 10^2^ μM) against fluorescent AGEs formation, being about 15 times more active than the reference drug aminoguanidine hydrochloride [177]. Further studies carried out with methanolic extracts from brown algae *Padina pavonica*, *Sargassum polycystum*, and *Turbinaria ornata*, rich in phlorotannins, inhibited the glucose-induced protein glycation and formation of protein-bound fluorescent AGEs (IC_50_ 15.16 μg/mL, 35.25 μg/mL and 22.7 μg/mL, respectively). Furthermore, brown algal extracts containing phlorotannins exhibited protective effects against AGEs formation in *Caenorhabditis elegans* (a species of nematode) with induced hyperglycemia [178]. From five phlorotannins isolated from *E. stolonifera*, only phlorofucofuroeckol-A inhibited, in a dose-dependent form, the induced non-enzymatic insulin glycation of D-ribose and D-glucose (IC_50_ 29.50 μM and 43.55 μM, respectively) [179]. These authors used computational analysis to find that phlorofucofuroeckol-A interacts with the Phe1 in insulin chain-B, blocking D-glucose access to the glycation site of insulin.

The need to secrete increasing amounts of insulin to compensate for progressive insulin resistance and the hyperglycemia-induced oxidative stress lead to an eventual deterioration of pancreatic β-cells [180]. Lee et al. [181] confirmed the protective effect of octaphlorethol A, a novel phenolic compound isolated from *Ishige foliacea*, against streptozotocin (STZ)-induced pancreatic β-cell damage investigated in a rat insulinoma cell line (RINm5F pancreatic β-cells). Thus, octaphlorethol A reduced the intracellular reactive oxygen species (ROS) and generation of thiobarbituric acid reactive substances (TBARs), extensively produced by STZ-treated pancreatic β-cells. The oxidative stress involved in diabetes-associated pathological damages reduces antioxidant enzyme activities (catalase (CAT), superoxide dismutase (SOD), and glutathione peroxidase (GPx)), and octaphlorethol A treatment increased the enzyme activity due to its antioxidant potency. The phlorotannins isolated from *E. cava*, 6,6-bieckol, phloroeckol, dieckol and phlorofucofuroeckol inhibited high glucose-induced ROS and cell death in zebrafish. Particularly, the antioxidant activity of dieckol significantly reduced heart rates, ROS, nitric oxide (NO) and lipid peroxidation generation in high glucose-induced oxidative stress. Dieckol also reduced overexpression of inducible nitric oxide synthase (iNOS) and cyclooxygenase-2 (COX-2) [182]. A recent study addressed the efficacy of an extract of the red seaweed *Polysiphinia japonica* on preserving cell viability and glucose-induced insulin secretion in a pancreatic β-cell line, Ins-1, treated with palmitate [183]. However, the tested extract contained, in addition to polyphenols, other components such as carbohydrates, lipid and proteins; hence, the described bioactivities may not be due only to polyphenols.

Glucose uptake and disposal mainly occurs in the skeletal muscle, playing an important role in the energy balance regulation [184]; marine polyphenols are also involved in this mechanism. Lee et al. [185] confirmed that octaphlorethol A from *Ishige foliacea* increased glucose uptake in skeletal muscle cells (differentiated L6 rat myoblast). Furthermore, this compound increased glucose transporter 4 (Glut4) translocation to the plasma membrane, in a process depending on the protein kinase B (Akt) and AMP-activated protein kinase (AMPK) activation, a therapeutic target for treatment of hyperglycemia, which is associated with insulin resistance [186]. 

**Table 4:** Effect of marine phenolics in the prevention of type 2 diabetes mellitus (T2DM).

Compounds/Marine Source	Test Model	Outcome	Ref.
Five isolated phlorotannins from *E. cava* (fucodiphloroethol G, dieckol, 6,6′-bieckol, 7-phloroeckol, phlorofucofuroeckol-A)	In vitro assay: α-glucosidase and α-amylase inhibitory activity	Inhibition of α-glucosidase (IC_50_ values ranged from 10.8 μM for dieckol to 49.5 μM for 7-phloroeckol) and α-amylase (IC_50_ values ranged from 125 μM for dieckol to <500 μM for the rest of compounds, except 7-phloroeckol with a value of 250 μM) activities	[165]
Methanolic extract isolated from *A. nodosum* rich in phlorotannins	In vitro assay: α-glucosidase and α-amylase inhibitory activity	Inhibition of α- glucosidase (IC_50_~20 μg/mL GAE) and α-amylase (IC_50_~0.1 μg/mL GAE) activities	[166]
Cold aqueous and ethanolic extracts of *A. nodosum* and *F. vesiculosus* rich in phlorotannins	In vitro assay: α-glucosidase and α-amylase inhibitory activity	Inhibition of α- glucosidase (IC_50_~0.32–0.50 μg/mL GAE for *F. vesiculosus*) and α-amylase (IC_50_~44.7–53.6 μg/mL GAE for *A. nodosum*) activities	[167]
Methanolic extract from *Alaria marginata* and *Fucus distichus* rich in phlorotannins	In vitro assay: α-glucosidase and α-amylase inhibitory activity	Inhibition of α- glucosidase (IC_50_~0.89 μg/mL) and α-amylase (IC_50_~13.9 μg/mL) activities	[168]
Polyphenol-rich extracts from *L. trabeculate*	In vitro assay: α-glucosidase and lipase activity	Inhibition of α-glucosidase and lipase activities (IC_50_ < 0.25 mg/mL)	[103]
Crude extract and semi-purified phlorotannins from *F. vesiculosus* composed by fucols, fucophlorethols, fuhalols and several other phlorotannin derivatives	In vitro assay: α-glucosidase, α-amylase and pancreatic lipase inhibitory activity	Inhibition of α-amylase (IC_50_~28.8–2.8 μg/mL), α-glucosidase (IC_50_~4.5–0.82 μg/mL) and pancreatic lipase (IC_50_~45.9–19.0 μg/mL) activities	[120]
Phlorotannin derivatives from *E. cava*	In vitro assay: α-glucosidase inhibitory activity	Inhibition of α-glucosidase activity (IC_50_~2.3–59.8 μM) Kinetic parameters of receptor–ligand binding	[163]
Phlorotannin-targeted extracts from four edible *Fucus* species (*F. guiryi*, *F. serratus*, *F. spiralis* and *F. vesiculosus*)	In vitro assay: α-glucosidase and α-amylase inhibitory activity	Inhibition of α-glucosidase (IC_50_~2.48–4.77 μg/mL), α-amylase (IC_50_~23.31–253.31 μg/mL) and xanthine oxidase (IC_50_~157.66–800.08 μg/mL) activities	[8]
Marine-derived bromophenol compound (3,4-dibromo-5-(2-bromo-3,4-dihydroxy-6-(ethoxymethyl)benzyl)benzene-1,2-diol) isolated from *Rhodomela confervoides*	In vitro: insulin resistant C2C12 cells treated with bromophenol (0.1–0.5 μM for phenol)	Inhibition of PTP1B activity (IC_50_~0.84 μM) Activation of insulin signaling and potentiate insulin sensitivity	[172]
3-Bromo-4,5-bis(2,3-dibromo-4,5-dihydroxybenzyl)-1,2-benzenediol isolated from the red alga *Rhodomela confervoides*	In vitro: palmitate-induced insulin resistance in C2C12 cells treated with bromophenol (0.5–2.0 μM for phenol)	Inhibition of PTP1B activity (IC_50_~2 μM) Activation of insulin signaling and prevent palmitate-induced insulin resistance	[173]
Phlorofucofuroeckol-A, eckol, phloroglucinol, fucofuroeckol A, dieckol and 8,8′-bieckol isolated and crude phlorotannins from *Lessoniaceae*	In vitro assay: human and bovine serum albumin models	Inhibition of AGEs formation, crude phlorotannins showed IC_50_~0.43–0.53 mg/mL, and among the purified phlorotannins, phlorofucofuroeckol A was the most active (IC_50_~4.1–4.8 μM)	[177]
Methanolic extract from *P. pavonica* and *Turbinaria ornate* rich in phlorotannins	In vitro assay: BSA-glucose assay In vivo: *Caenorhabditis elegans* with induced hyperglycemia	Inhibition of AGEs formation (IC_50_~15.16 μg/mL, 35.25 μg/mL and 22.70 μg/mL, respectively) Inhibition of AGEs formation	[178]
Phlorofucofuroeckol-A isolated from *E. stolonifera*	In vitro assay for non-enzymatic insulin glycation	Inhibition of AGEs formation (IC_50_ 29.50–43.55 μM for D-ribose and D-glucose-induced insulin glycation, respectively)	[179]
Octaphlorethol A isolated from *Ishige foliacea*	In vitro: STZ-induced pancreatic β-cell damage (RINm5F pancreatic β-cells) (12.5–50.0 μg/mL for phenol)	Decreased the death of STZ-treated pancreatic β-cells Decreased the TBARS and ROS Increased the activity of antioxidant enzymes	[181]
6,6-Bieckol, phloroeckol, dieckol and phlorofucofuroeckol isolated from *E. cava*	In vivo: high glucose-stimulated oxidative stress in Zebrafish, a vertebrate model (10–20 μM of phenols)	Inhibition of high glucose-induced ROS and cell death Dieckol reduced the heart rates, ROS, NO and lipid peroxidation Dieckol reduced the overexpression of iNOS and COX-2	[182]
Extract isolated from the red seaweed *Polysiphonia japonica*	In vitro: palmitate-induced damage in β-cells (Ins-1 cells) (1–10 μg/mL of extract)	Inhibited the palmitate-induced damage in β-cells Preserved the glucose-induced insulin secretion in β-cells	[183]
Octaphlorethol A from *Ishige foliacea*	In vitro: rat myoblast L6 cells (6.25–50 μM of phenol)	Increased the glucose uptake Increased the Glut4 translocation to the plasma membrane, via Akt and AMPK activation	[185]
Dieckol *isolated* from *E. cava*	In vivo: STZ-induced diabetic mice (acute, 100 mg/kg bw of dieckol administered orally)	Delayed the absorption of dietary carbohydrates	[187]
2,7’’-Phloroglucinol-6,6’-bieckol from *E. cava*	In vivo: STZ-induced diabetic mice (acute, 10 mg/kg bw of phenol administered orally)	Delayed the absorption of dietary carbohydrates Inhibition of α-glucosidase and α-amylase activities (IC_50_ 23.35 μM and 6.94 μM, respectively)	[188]
Polyphenol-rich seaweed extract from *F. vesiculosus*	In vivo: 38 healthy adults (acute, 500 mg and 2000 mg of phenol)	No change in postprandial blood glucose and insulin levels	[189]
Dieckol isolated from brown seaweed *E. cava*	In vivo: a T2DM mouse model (C57BL/KsJ-db/db) (10 and 20 mg/kg bw of phenol for 14 days administered intraperitoneal injection)	Diminished the fasting blood glucose and insulin levels Diminished the body weight Decreased the TBARS Increased the activity of antioxidant enzymes in liver tissues Increased the levels of AMPK and Akt phosphorylation in muscle tissues	[190]
Polyphenol-rich extracts from brown macroalgae *L. trabeculata*	In vitro assay: α-glucosidase and lipase inhibitory activities --- In vivo: high-fat diet and STZ-induced diabetic rats (200 mg/kg/day bw of phenol for 4 weeks by gavage)	Inhibition of α-glucosidase and lipase activities (IC_50_ < 0.25 mg/mL) --- Diminished the fasting blood glucose and insulin levels Improved the serum lipid profile Improved the antioxidant stress parameters	[103]
Water-ethanolic extract of green macroalgae *Enteromorpha prolifera* rich in flavonoids	In vivo: STZ-induced diabetic rats (150 mg/kg/day bw of phenol for 4 weeks by gavage)	Diminished the fasting blood glucose and improved oral glucose tolerance Hypoglycemic effect by increasing IRS1/PI3K/Akt and suppressing JNK1/2 in liver	[191]
Dieckol-rich extract of brown algae *E. cava*	In vivo: 8 pre-diabetic adults (1500 mg per day for 12 weeks)	Decreased the postprandial glucose, insulin, and C-peptide levels	[192]

GAE: gallic acid equivalents; PTP1B: protein tyrosine phosphatase 1B; AGEs: advanced glycation end-products; ROS: reactive oxygen species; TBARs: thiobarbituric acid reactive substances; NO: nitric oxide; iNOS: inducible nitric oxide synthase; COX-2: cyclooxygenase-2; Glut4: glucose transporter 4; Akt: protein kinase B; AMPK: AMP-activated protein kinase; PI3K: phosphoinositide 3-kinase; IRS1: Insulin receptor substrate 1; JNKs: c-Jun N-terminal kinases.

Since postprandial blood glucose is a stronger predictor of cardiovascular events than fasting blood glucose in T2DM [193], polyphenol-rich extracts from seaweeds have been evaluated for their postprandial effect. After oral administration of soluble starch with dieckol (100 mg/kg bw), isolated from *E. cava*, a significant reduction in the postprandial blood glucose level in both normal mice and STZ-induced diabetic mice [187] were observed. Likewise, a phlorotannin constituent of *E. cava* (2,7″-phloroglucinol-6,6’-bieckol) inhibited α-glucosidase and α-amylase activities (IC_50_ values of 23.35 and 6.94 μM, respectively), which was more effective than that observed with the positive control acarbose (IC_50_ values of 130.04 and 165.12 μM, respectively). In addition, this phlorotannin alleviated postprandial hyperglycemia in diabetic mice treated with 10 mg/kg bw [188]. A randomized cross-over trial carried out by Murray et al. [189] evaluated the impact of a single dose of a polyphenol-rich seaweed extract from *F. vesiculosus* on postprandial glycemic control in 38 healthy adults. Neither low (500 mg) nor high (2000 mg) doses of the polyphenol-rich brown seaweed affected the postprandial blood glucose and insulin levels in healthy volunteers.

The in vivo chronic treatment with polyphenol-rich extracts from seaweeds showed an important activity in the attenuation of T2DM. The antidiabetic activity of dieckol isolated from brown seaweed *E. cava* was evaluated in a T2DM mouse model (C57BL/KsJ-db/db). Dieckol was administrated daily at doses of 10 and 20 mg/kg bw for 14 days. Results showed a significant reduction in blood glucose and serum levels as well as body weight, when compared to the untreated group [190]. Furthermore, reduced TBARs and increased activity of antioxidant enzymes (SOD, CAT and GPx) in liver tissues, as consequence of the antioxidant potency of phlorotannins, and increased levels of AMPK and Akt phosphorylation in muscle tissues, which play a vital role in the glucose homeostasis, were observed in the dieckol treated group. Another recent study showed the capacity of a polyphenol-rich extracts from the brown macroalgae *Lessonia trabeculata* to attenuate hyperglycemia in high-fat diet and STZ-induced diabetic C57BL/6J rats treated for 4 weeks (200 mg/kg bw/day). Lower fasting blood glucose and insulin levels, as well as a better serum lipid profile and antioxidant stress parameters compared with the diabetic control group, were observed [103]. Similarly, a water-ethanolic extract of green macroalgae *E. prolifera* rich in flavonoids showed antidiabetic activity, by improving oral glucose tolerance and insulin sensitivity, decreasing fasting blood glucose levels and protecting kidney and liver from high sucrose–fat diet on STZ-induced diabetic mice treated with 150 mg/kg bw/day of the assayed extract for 4 weeks. This flavonoid-rich fraction revealed a hypoglycemic effect as confirmed by activation of the IRS1/PI3K/Akt and inhibition of the c-Jun N-terminal kinases (JNK)1/2 insulin pathway in liver [191].

In pre-diabetic human subjects, the efficacy and safety of a dieckol-rich extract from *E. cava* was evaluated by the development of a double-blind, randomized, placebo-controlled clinical trial. The daily consumption of 1500 mg of the dieckol-rich extract decreased postprandial glucose, insulin, and C-peptide levels after 12 weeks, but there was no significant difference between the supplemented and placebo groups [192]. 

**Figure 2:**
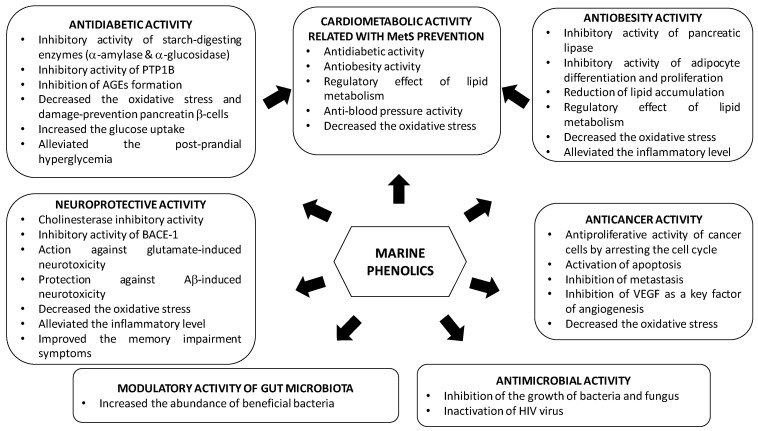
Mechanisms of action of marine phenolics.

Strong evidence regarding the antidiabetic activity of several marine polyphenols have been obtained (Table 4). They are involved in different and complementary mechanisms (Figure 2), although most of the studies are in vitro or in vivo with animals, not with humans as desirable. 

### 3.2. Obesity

Obesity—defined as the excessive or abnormal accumulation of body fat in the adipose tissue, energy imbalance, and lipogenesis—results from modern lifestyles characterized by high intakes of fat, sugar, and calories, in addition to poor exercise and physical activity [194]. The molecular mechanism of obesity mediated by cytokines, adiponectin, and leptin has been correlated with increasing inflammation and oxidative stress, and leads to the development of metabolic diseases including certain types of cancer, hyperglycemia, T2DM, high blood pressure, as well as liver, heart, and gallbladder diseases [195,196,197]. Consequently, researchers have been exploring functional materials of plant origin that contain antioxidants and other properties to combat obesity and its comorbidities, as an alternative to conventional approaches such as surgery and antiobesity drugs.

Pancreatic lipase is a key enzyme for triglyceride absorption in the small intestine, which hydrolyses triglycerides into glycerol and fatty acids. Pancreatic lipase inhibitors hinder fat digestion and absorption and are a potential therapeutic target for the treatment of diet-induced obesity in humans. In an ongoing search for new pancreatic lipase inhibitors from natural sources, polyphenols isolated from seaweeds have repeatedly shown inhibitory activity against this enzyme, such as a methanolic extract of the marine brown algae *E. bicyclis*. Bioassay-guided isolation of this methanolic extract using a pancreatic lipase inhibitory test led to the identification of six known phlorotannins: eckol, fucofuroeckol A, 7-phloroeckol, dioxindehydroeckol, phlorofucofuroeckol A, and dieckol. Among them, fucofuroeckol and 7-phloroeckol showed the most potent inhibitory effect on pancreatic lipase activity (IC_50_ values of 37.2 and 12.7 μM, respectively) [198]. More recently, Austin et al. [199] evaluated the inhibitory lipase activity of a polyphenol-rich extract from the edible seaweed *A. nodosum*. This crude extract showed higher inhibitory activity than the known commercial product, Orlistat. Additionally, a phlorotannin-enriched fraction obtained from the crude extract was even more potent than the un-purified extract. Although the purified extract also contained polysaccharides such as alginate that might contribute to the total inhibitory lipase activity, apparently these were less effective than phlorotannins (Table 5).

Obesity is related to adipogenesis, which is the process of pre-adipocyte differentiation into adipocytes. This process plays a central role in keeping lipid homeostasis and energy balance, by storing triglycerides (TG) and releasing free fatty acids in response to changing energy demands. Adipogenesis is regulated by multiple processes, including pre-adipocytes proliferation, differentiation, as well as fatty acid oxidation and synthesis, which are controlled by several factors [196,200]. Thus, the inhibitory effect of adipocyte differentiation and proliferation has been suggested to be an important strategy for preventing or treating obesity. Dieckol from *E. cava* [201], phloroglucinol, eckol, dieckol, dioxinodehydroeckol, and phlorofucofuroeckol A from *E. stolonifera* [202] and 6,6′-bieckol, 6,8′-bieckol, 8,8′-bieckol, dieckol and phlorofucofuroeckol A from *E. bicyclis* [203] exhibited antiobesity activity by suppressing the differentiation of 3T3-L1 pre-adipocytes cells in a dose-dependent manner. These phlorotannins were able to down-regulate the expression of the proliferator activated receptor gamma (PPARγ) and the CCAAT/enhancer-binding protein alpha (C/EBPα) [201,202,203]. The activation of C/EBPα promote differentiation of preadipocytes through cooperation with PPARγ resulting in transactivation of adipocyte-specific genes such as fatty acid binding protein (FABP) and fatty acid synthase (FAS). Sterol regulatory element-binding protein 1 (SREBP1) is the earliest transcription factor, which also appears to be involved in adipocyte differentiation, and increases the expression of several lipogenic genes, including acyl-coA carboxylase (ACC) and FAS. Therefore, over expression of these transcription factors can accelerate adipogenesis. In this sense, dieckol, moreover, down-regulated the expression of the SREBP1 and that of the FABP4 by AMPK activation [201], the latter also related to the obesity control. In addition, 6′6′-bieckol down-regulated the sterol regulatory element binding protein-1c (SREBP-1c), the FAS and the ACC [203]. Similarly, Kong et al. [204] characterized the antiadipogenic activity of triphlorethol-A, eckol and dieckol isolated from *E. cava* in differentiating 3T3-L1 pre-adipocytes by measuring glycerol release level and adipogenic-related gene expression. These phlorotannins increased the glycerol secretion and reduced the glucose consumption level of adipocytes. In addition, phlorotannins down-regulated the expression of PPARγ, C/EBPα, SREBP-1c, as well as FABP4, FAS, acyl-CoA synthetase-1 (ACS1), fatty acid transport protein-1 (FATP1) and leptin. FATP1 has been reported to take part in fatty acid utilization along with FABP4 [205]. Leptin is a hormone related to food intake and body weight reduction. Obese subjects present leptin resistance, i.e., despite this enzyme being found in high levels in these subjects, it is unable to exercise any anorexigenic effect [206]. Phlorotannins also increased mRNA expression of hormone-sensitive lipase while they suppressed perilipin and tumor necrosis factor alpha (TNFα) expressions. Kim et al. [207] demonstrated that an extract containing eckol, dieckol and phlorofucofuroeckol-A from *E. cava* inhibited adipogenesis in 3T3-L1 adipocytes, shown by the significant reduction in glucose utilization and TG accumulation without showing cytotoxicity. This suppressive effect may be mediated by decreasing the expression levels of C/EBPα, SREBP-1c, adipocyte fatty acid binding protein (A-FABP), FAS and adiponectin (Table 4). Karadeniz et al. [208] also confirmed the antiadipogenic effect of triphlorethol-A, eckol and dieckol isolated from *E. cava* on 3T3-L1 pre-adipocytes, by reducing lipid accumulation and suppressing the expression of adipogenic differentiation markers. Considering that adipocytes and osteoblasts are derived from a common mesenchymal stem cell precursor, molecules that lead to osteoblastogenesis inhibit adipogenesis and vice versa. Thus, these authors also observed that the isolated phlorotannins successfully enhanced the osteoblast differentiation evaluated in MC3T3-E1 pre-osteoblasts, by increasing the alkaline phosphatase activity along with raising the osteoblastogenesis indicators and intracellular calcification. These results showed the potential of the selected phlorotannins for mitigating obesity and osteoporosis, which are closely related [208]. 

A complete study developed by Choi et al. [209] demonstrated that dieckol, a major phlorotannin in *E. cava*, suppressed lipid accumulation in 3T3-L1 cells, high-fat diet-fed zebrafish and mice (15 mg/kg bw/day and 60 mg/kg bw/day for 11 weeks). Furthermore, the findings suggested that dieckol was able to inhibit early adipogenic events by suppressing cell cycle progression, and played an important role in regulating AMPKα, ERK, and Akt signaling pathways to inhibit lipid accumulation. Recently, Ko et al. [210] demonstrated that the treatment of 3T3-L1 pre-adipocytes with 5-bromo-3,4-dihydroxybenzaldehyde isolated from the red alga *Polysiphonia morrowii* could inhibit intracellular lipid accumulation and TG levels by downregulating protein expression of adipogenic-specific factors such as PPARγ, C/EBPα, SREBP-1, FABP4, FAS, leptin, and adiponectin through phosphorylation of AMPK and ACC (Table 5).

Visceral obesity is characterized by chronic local and systemic inflammation [195]. It is well established that an increase in pro-inflammatory cytokines may be related to enlarged adipose tissue, and dysregulation of lipid metabolism, ultimately leading to insulin resistance. Thus, a phlorotannin fraction from the brown alga *Fucus distichus* decreased mRNA expression of acute and chronic inflammatory biomarkers via TRL attenuation in RAW 264.7 macrophages. Additionally, *F. distichus* fractions decreased lipid accumulation in 3T3-L1 adipocytes up to 55% and increased free glycerol concentrations, by increasing in adiponectin and uncoupling protein 1 (UCP-1) and decreasing in leptin mRNA expression [211]. Compared with lean adipocytes, hypertrophic adipocytes had higher expression of inflammatory cytokines (e.g., TNFα, interleukin (IL)-1β) and of receptors for advanced glycation end-products (RAGEs) and RAGE ligands (e.g., AGE, HMGB1, S100b, free fatty acids (FFAs)) [212]. Choi et al. [213] demonstrated the antiobesity effect of pyrogallol-phloroglucinol-6,6-bieckol (PPB) contained in *E. cava* by reducing the expression of RAGE and the secretion of ligands in a mouse model of diet-induced obesity that consumed PPB (2.5 mg/kg bw/day) for 4 weeks. In addition, this phlorotannin reduced the number of activated macrophages and inflammatory cytokine levels (TNFα and IL-1β).

The regulatory effect of marine phenolics on lipid metabolism has also been evaluated. Typically, dyslipidemia of obesity consists of increased fasting plasma TG and FFAs, decreased HDL-cholesterol (HDL-C) and normal or slightly increased LDL-cholesterol (LDL-C). Yeo et al. [214] demonstrated that oral administration of polyphenol extracts of the marine brown algae *E. cava* and dieckol effectively suppressed body weight gain and reduced total cholesterol (TC), TG and LDL-C levels in high-fat diet-induced obese mice treated with 1.25, 2.5 and 5.0 mg extract/mouse or 0.5, 1.0 and 2.0 mg dieckol/mouse for 4 weeks. The antihyperlipidemic effect was related to the inhibition of 3-hydroxyl-methyl glutaryl coenzyme A (HMGCoA) reductase activity, which is involved in the metabolic pathway that produces cholesterol and other isoprenoids. Likewise, Park et al. [215] confirmed the antiobesity activity of a polyphenol-rich fraction of the brown alga *E. cava* in high fat diet-induced obese mice. Oral administration of a polyphenol extract (200 mg/kg bw/day) for 8 weeks was effective in reducing body weight gain, body fat, and hyperglycemia, as well as in improving glucose tolerance. The mRNA expression of inflammatory cytokines (TNF-α and IL-1β) and macrophage marker gene (F4/80) was decreased in treated obese mice. These authors compared the efficacy of *E. cava* from different areas in Korea: that from Gijang was consistently more effective than that from Jeju due to its higher amounts of polyphenols and richness in 8,8′-bieckol, the major component in Gijang extract. In agreement with Park et al. [215], a later study developed by Eo et al. [216] reported that the treatment with a polyphenol-rich extract of *E. cava* containing dieckol, 2,7”-phloroglucino-6,6′-bieckol, pyrogallo-phloroglucinol-6,6′-bieckol and phlorofucofuro-eckol A (100 mg/kg bw/day or 500 mg/kg bw/day, 5 times a week for 12 weeks) was able to reduce body weight gain, adipose tissue mass, plasma lipid levels (TC and TG), hepatic fat depositions, insulin resistance and plasma leptin/adiponectin ratio of diet-induced obese mice. Moreover, polyphenol supplementation selectively ameliorated hepatic protein levels associated with hepatic lipogenesis (SREBP-1c, PPARα, FAS, and LPL), fatty acid β-oxidation (p-ACC and CPT1A), inflammation (TNF-α, IL-1β and NFkB) as well as enhancing the antioxidant defense system by activating the AMPK and SIRT1 signaling pathway (Table 4). Ding et al. [217] demonstrated the strong effect of diphlorethohydroxycarmalol, the most abundant bioactive compound in *Ishige okamurae*, against high-fat levels in diet-induced obese mice through in vivo regulation of multiple pathways. Oral administration of this polyphenol (25 and 50 mg/kg bw/day for six weeks) significantly reduced adiposity and body weight gain and improved lipid profile (lowered TG and LDL-C and increased HDL-C levels). This compound reduced hepatic lipid accumulation, by the reduction in expression levels of the critical enzymes for lipogenesis (SREBP-1c, FABP4, and FAS). In addition, diphlorethohydroxycarmalol reduced the adipocyte size and the expression levels of key adipogenic-specific proteins and lipogenic enzymes such as PPARγ, C/EBPα, SREBP-1c, FABP4, and FAS, which regulate the lipid metabolism in the epididymal adipose tissue. Finally, diphlorethohydroxycarmalol stimulated the phosphorylation of AMPK and ACC in both liver and epididymal adipose tissue. 

Clinical trials also have demonstrated the potential of marine phenolics to prevent obesity. The efficacy of a polyphenol-rich extract from *E. cava* (low dose-72 mg/day or high dose-144 mg/day) was tested in 97 overweight adults enrolled in a randomized, double-blind, placebo-controlled clinical trial with parallel-group design. Results demonstrated that the polyphenol-rich extract consumed for 12 weeks lowered body fat and serum lipid levels (TC and LDL-C) [218].

All these data together highlight the potential of marine phenols in the prevention and treatment of obesity (Table 5, Figure 2), although more studies, especially clinical trials, would reinforce their use in the management of obesity.

**Table 5:** Effect of marine phenolics in the prevention of obesity.

Compounds/Marine Source	Test Model	Outcome	Ref.
Methanolic extract of *E. bicyclis* (eckol, fucofuroeckol A, 7-phloroeckol, dioxindehydroeckol, phlorofucofuroeckol A, and dieckol)	In vitro: assay of pancreatic lipase activity	Inhibition of pancreatic lipase activity; fucofuroeckol A and 7-phloroeckol were the most potent (IC_50_ values of 37.2 and 12.7 μM, respectively)	[198]
Polyphenol-rich extract (crude) from the edible seaweed *A. nodosum* and phlorotannin-enriched fraction from crude extract	In vitro: assay of pancreatic lipase activity	Inhibition of pancreatic lipase activity Evaluated the interaction between phlorotannins and polysaccharides on inhibitory lipase activity and phlorotannins were more effective	[199]
Dieckol isolated from *E. cava*	In vitro: 3T3-L1 pre-adipocytes cells (25–100 μM of phenol)	Suppression of pre-adipocytes differentiation Down-regulated the expression of PPARγ, C/EBPα, SREBP1 and FABP4 by AMPK activation	[201]
Phloroglucinol, eckol, dieckol, dioxinodehydroeckol, and phlorofucofuroeckol A isolated from *E. stolonifera*	In vitro: 3T3-L1 pre-adipocytes cells (12.5–100 μM of phenol)	Suppression of pre-adipocytes differentiation Down-regulated the expression of PPARγ and C/EBPα	[202]
6,6′-Bieckol, 6,8′-bieckol, 8,8′-bieckol, dieckol and phlorofucofuroeckol A isolated from *E. bicyclis*	In vitro: 3T3-L1 pre-adipocytes cells (10–50 μg/mL of phenol)	Suppression of pre-adipocytes differentiation Down-regulated the expression of PPARγ, C/EBPα, SREBP-1c, FAS and ACC	[195]
Triphlorethol-A, eckol and dieckol from *E. cava*	In vitro: 3T3-L1 pre-adipocytes cells (5 μM of phenol)	Increased the glycerol secretion and reduced glucose consumption level Down-regulated the expression of PPARγ, C/EBPα, SREBP-1c as well as FABP4, FATP1, FAS, leptin and ACSL1	[204]
Extract from *E. cava* containing eckol, dieckol and phlorofucofuroeckol-A	In vitro: 3T3-L1 adipocytes cells (50 μg/mL of extract)	Inhibited the glucose utilization and TG accumulation Down-regulated the expression of C/EBPα, SREBP-1c, A-FABP, FAS and adiponectin	[207]
Triphlorethol-A, eckol and dieckol isolated from *E. cava*	In vitro: 3T3-L1 pre-adipocytes cells (1–20 μM of phenol) --- In vitro: MC3T3-E1 cells (1–20 μM of phenol)	Suppressed the lipid accumulation and expression of adipogenic differentiation markers --- Enhanced the osteoblast differentiation by increasing alkaline phosphatase activity and raising intracellular calcification	[208]
Dieckol from *E. cava*	In vitro: 3T3-L1 pre-adipocytes cells (25–100 μM of phenol) --- In vivo: high-fat diet-fed zebrafish (1–4 μM of phenol) --- In vivo: high-fat diet-fed mice (15 mg/kg bw/day or 60 mg/kg bw/day, 11 weeks administered orally)	Suppressed the lipid accumulation in the three models Inhibited the early adipogenic events by suppressing cell cycle progression Regulated the AMPKα, ERK, and Akt signaling to inhibit lipid accumulation	[209]
5-Bromo-3,4-dihydroxybenzaldehyde isolated from *Polysiphonia morrow*	In vitro: 3T3-L1 pre-adipocytes cells (25–100 μM)	Inhibited the intracellular lipid accumulation and triglyceride levels Down-regulated the expression of PPARγ, C/EBPα, SREBP-1, FABP4, FAS, leptin, and adiponectin by AMPK and ACC activation.	[210]
Phlorotannin fraction from *Fucus distichus*	In vitro: murine macrophage RAW 264.7 cells (12.5–50 μg/mL of extract) --- In vitro: 3T3-L1 adipocytes cells (12.5–50 μg/mL of extract)	Anti-inflammatory activity via TLR attenuation in macrophages -- Decreased the lipid accumulation in 3T3-L1 adipocytes cells	[211]
Pyrogallol-phloroglucinol-6,6-bieckol from *E. cava*	In vivo: mouse model of diet-induced obesity (2.5mg/kg bw/day for 4 weeks administered orally)	Reduced the expression of RAGE and the secretion of ligands Reduced the inflammatory cytokine level (TNFα and IL-1β)	[213]
Polyphenol extracts from *E. cava* and dieckol	In vivo: high-fat diet-induced obese mice (5.0 mg, 2.5 mg and 1.25 mg extract/mouse; 2.0 mg, 1.0 mg and 0.5 mg dieckol/mouse for 4 weeks administered orally) --- In vitro: 3T3-L1 pre-adipocytes cells	Suppressed the body weight gain Reduced the TC, TG and LDL-C levels ---- Inhibited the lipid accumulation Inhibition of HMGCoA reductase activity	[214]
Polyphenol-rich fraction of *E. cava* from Gijang (Korea)	In vivo: high-fat diet-induced obese mice (200 mg/kg bw for 8 weeks by oral intubation)	Reduced the body weight gain, body fat and hyperglycemia Reduced the mRNA expression of inflammatory cytokines (TNF-α and IL-1β) and macrophage marker gene (F4/80)	[215]
Polyphenol-rich fraction of *E. cava* containing dieckol, 2,7”-phloroglucino-6,6′-bieckol, pyrogallo-phloroglucinol-6,6′-bieckol and phlorofucofuro-eckol A	In vivo: high-fat diet-induced obese mice (100mg/kg bw/day or 500 mg/kg bw/day, 5 times a week for 12 weeks by gavage)	Reduced the body weight gain, body fat, plasma lipid levels (TC and TG), insulin resistance and plasma leptin/adiponectin ratio Ameliorated the hepatic protein levels: hepatic lipogenesis (PPARγ, SREBP-1c, FAS and LPL), fatty acid β-oxidation (p-ACC and CPT1A), inflammation (TNF-α, NFkB and IL-1β) and antioxidant defense system	[216]
Diphlorethohydroxycarmalol isolated from *Ishige okamurae*	In vivo: high-fat diet-induced obese mice (25 mg/kg bw/day or 50 mg/kg bw/day for 6 weeks administered orally)	Reduced the body weight gain, body fat and hepatic lipid accumulation, and improved lipid profile Reduced the hepatic lipid accumulation by reduction in expression level of SREBP-1c, FABP4, and FAS Reduced the adipocyte size by down-regulation of enzyme expression (PPARγ, C/EBPα, SREBP-1c, FABP4, and FAS)	[217]
Polyphenol-rich extract from *E. cava*	In vivo: 97 overweight adults (low dose-72 mg/day or high dose-144 mg/day for 12 weeks)	Decreased the body fat and serum lipoid levels (TC and LDL-C)	[218]

PPARγ: proliferator activated receptor gamma; C/EBPα: CCAAT/enhancer-binding protein alpha; SREBP1: sterol regulatory element binding protein 1; FABP4: fatty acid binding protein 4; AMPK: AMP-activated protein kinase; SREBP-1c: sterol regulatory element binding protein-1c; FAS: fatty acid synthase; ACC: acyl-CoA carboxylase; FATP1: fatty acid transport protein-1; ACSL1: adipose acyl-CoA synthetase 1; ERK: extracellular signal-regulated kinase; Akt: protein kinase B; TLR: toll-like receptor; RAGE: receptor for advanced glycation end-products; TNFα: tumor necrosis factor alpha; IL-1β: interleukin 1b; TC: total cholesterol; TG: triglycerides; LDL-C: LDL-cholesterol; HMGCoA: 3-hydroxyl-methyl glutaryl coenzyme A; ACC: acetyl-CoA carboxylase; CPT1A: carnitine palmitoyltransferase I; NFkB: nuclear factor kappa B.

### 3.3. Metabolic Syndrome.

Metabolic syndrome (MetS) is not a disease but a metabolic disorder that includes hypertension, obesity, glucose dysregulation and dyslipidemia [219]. A person has MetS when three or more of the following five cardiovascular risk factors have been diagnosed: (i) central obesity (waist circumference: men ≥102 cm; women ≥88 cm); (ii) elevated TG (≥150 mg/dL); (iii) diminished HDL-C (men <40 mg/dL; women <50 mg/dL) (or treated for dyslipidemia); (iv) systemic hypertension (≥130/≥85 mm Hg) (or treated for hypertension); (v) elevated fasting glucose (≥100 mg/dL) (or treated for hyperglycemia) [220]. MetS appears to be two times more frequent in women than in men, and menopause contributes to its rapid acceleration [221]. A recent study examined prospectively the association between habitual dietary iodine and seaweed consumption and the incidence of MetS among 2588 postmenopausal women 40 years or older in the Korean Multi-Rural Communities Cohort (MRCohort) [222] for an average time of 2.85 years (between 2 and 4 years). Results showed an inverse association between seaweed consumption with MetS incidence. The unmeasured bioactives of seaweed, such as polysaccharides, peptides, carotenoids and polyphenols, make it difficult to understand the real involvement of marine phenolics in the observed effects (Table 6).

**Table 6:** Effect of marine phenolics in the prevention of metabolic syndrome (MetS).

Compounds/Marine Source	Test Model	Outcome	Ref.
Dietary iodine and seaweed consumption	In vivo: 2588 postmenopausal women for 2.85 years (between 2 and 4 years)	Inverse association between seaweed consumption with MetS incidence	[222]
Bioactive fraction of *Sargassum wightii*	In vitro: assays of ACE enzyme activity and antioxidant activity (DPPH, ABTS and FRAP)	Inhibition of ACE activity (IC_50_ 56.96 μg/mL) and improved the antioxidant potency determined	[223]
An extract l from *E. cava* and pyrogallol-phloroglucinol-6,6-bieckol	In vivo: two mice models, high-fat diet-induced obese mice and high-cholesterol and saline diet-induced hypertension mice (70 mg extract or 500 mg extract or 2.5 mg pure phenol/kg bw/day for 4 weeks administered orally) In vitro: VSMC cells, an endothelial cell line	Reduced the blood pressure and serum lipoprotein levels in vivo Reduced the adhesion molecule expression, endothelial cell death and excessive migration and proliferation of VSMCs in vitro, as well as in the obese and hypertension mouse models	[224]
Ethanolic extract from *Ulva lactuca* enriched in phlorotannins	In vivo: hypercholesterolemic mice (250 mg/kg body weight for 4 weeks by gavage)	Improved the heart oxidative stress, plasma biochemical parameters and index of atherogenesis Down-regulated the expression of pro-inflammatory cytokines (TNFα, IL-1β and IL-6) in the heart	[225]
Food supplement from *K. alvarezii*	In vivo: rats fed for 8 weeks on high-carbohydrate, high-fat diet, alone or supplemented with 5% (*w/w*) algae	Reduced the body weight, adiposity, systolic blood pressure and plasma lipid levels Improved the heart and liver structure	[226]

ACE: angiotensin-I converting enzyme; DPPH: 2,2-diphenyl-1-picrylhydrazyl: ABTS: 2,2′-azino-bis (3-ethylbenzothiazoline-6-sulphonic acid); FRAP: ferric reducing antioxidant power: VSMC: human vascular smooth muscle cell line; TNFα: tumor necrosis factor alpha; IL-1β: interleukin 1β; IL-6: interleukin 6.

The aforementioned in vitro and in vivo studies have evidenced the involvement of marine phenolics in regulating lipid metabolism, hyperglycemia and obesity; these studies were reviewed by Gomez-Guzman et al. [227]. Additionally, hypertension, which is a strong independent risk factor for stroke and coronary heart disease, is also a cardiovascular risk factor in patients with MetS [228]. Angiotensin-I converting enzyme (ACE) is a zinc-containing metalloproteinase that catalyzes the conversion of angiotensin I to angiotensin II, a potent vasoconstrictor involved in the pathogenesis of hypertension. ACE also facilitates the degradation of the vasodilator bradykinin. This enzyme has a crucial role in the control of blood pressure and its inhibition has become a major target for hypertension control. Seca et al. [229] recently reviewed several marine polyphenols that have been reported to inhibit ACE activity. A bioactive fraction of the brown algae *Sargassum wightii* with optimal antioxidant and ACE inhibition activities (IC_50_ 56.96 μg/mL) was characterized by Vijayan et al. [223]. An in vivo study evaluated the efficacy of a polyphenol-rich extract from *E. cava* as well as its major component (pyrogallol-phloroglucinol-6,6-bieckol) for improving blood circulation in diet-induced obese and diet-induced hypertension mouse models [224]. After four weeks of administering 70 mg and 500 mg of extract/kg bw or 2.5 mg of phenol/kg bw, the study found a reduction in blood pressure and in serum lipoprotein levels in the obese and hypertension mouse models. A reduced expression of adhesion molecules and endothelial cell death as well as a reduction in excessive migration and proliferation of vascular smooth cells was also observed (Table 6).

In vivo studies with animals supplemented with diet-induced MetS have also evidenced the potential of seaweed polyphenols to prevent metabolic disorders. An ethanolic extract from *U. lactuca* enriched in phlorotannins was tested against hypercholesterolemia and other risk factors involved in CVD. Treatment of hypercholesterolemic mice with *U. lactuca* extract (250 mg/kg body weight) for 4 weeks revealed a cardioprotective effect by improving heart oxidative stress, plasma biochemical parameters, and index of atherogenesis. Additionally, a reduction in gene expression of proinflammatory cytokines (TNFα, IL-1β and IL-6) in the heart of *U. lactuca*-supplemented animals was also observed [225]. *Kappaphycus alvarezii*, a red seaweed, was tested as a food supplement to prevent diet-induced MetS in rats. Rats were randomly divided and fed for 8 weeks with control diet or high-fat/high-carbohydrate diet supplemented with 5% (*w/w*) algae. *Kappaphycus*-treated rats showed normalized body weight and adiposity, lower systolic blood pressure, improved heart and liver structure, and lower plasma lipids [226]. The hypotensive activity of marine polyphenols, in addition to their antidiabetic, antilipidemic and antiobesity activities, turns this group of compounds into allies to combat MetS and related cardiovascular complications (Table 6 and Figure 2).

### 3.4. Neurodegenerative Diseases

Seaweed-derived phenols have been described to possess neuroprotective properties [230]. Although this pathology has been less explored than those described above, knowing the role of phenolic constituents of seaweed as neuro-active compounds has gained tremendous interest in the last decade. Alzheimer’s disease (AD) is the most common form of irreversible dementia, and its neuropathological hallmarks are characterized by amyloid plaques and neurofibrillary tangles composed of aggregated amyloid-β peptides (Aβ) and microtubule-associated protein tau, respectively [231]. Although the exact mechanisms of Aβ-induced neurotoxicity are still unclear, it has been reported that pathological deposition of Aβ leads to cholinergic dysfunction, glutamate excitotoxicity, beta-amyloid aggregation, oxidative stress, apoptosis and neuro-inflammation, inducing the progressive degeneration of *cognitive functions* in AD patients (Figure 2).

AD development has been linked with an impaired cholinergic pathway which is caused by upregulation of acetylcholinesterase (AChE) and butyrylcholinesterases (BChE) as well as rapid depletion of acetylcholine (AChE) [232]. In addition, BACE-1 (β-site amyloid precursor protein cleaving enzyme 1) is the major β-secretase for generation of Aβ by neurons [233] and its inhibition could block one of the earliest pathologic events in AD. The activity of some phlorotannins, in particular eckols from *E. cava* [234] and *E. bicyclis* [235], showed an inhibitory effect against AChE and BChE activities, higher than the currently used anti-AD drugs. Recently, aqueous extracts of some seaweeds (*G. beckeri*, *G. pristoides*, *Ulva rigida* and *Ecklonia maxima*), composed mainly by phloroglucinol, catechin and epicatechin 3-glucoside, showed high antioxidant potency, inhibitory activity of AChE and BChE enzymes and Aβ aggregation [236] (Table 7). The study by Olasehinde et al. [237] revealed that aqueous-ethanolic extracts of *G. pristoides*, *E. maxima*, *U. lactuca* and *G. gracilis* containing phlorotannins, flavonoids and phenolic acids exhibited a strong inhibitory activity of BACE-1, AChE and BChE enzymes, as well as hampered Aβ aggregation. Choi et al. [234] showed that phlorofucofuroeckol isolated from *E. cava* also reduced BACE-1 activity (IC_50_ values in Table 7).

**Table 7:** Effect of marine phenolics in the prevention of Alzheimer’s disease (AD).

Compounds/Marine Source	Test Model	Outcome	Ref.
Phlorotannin-rich extract from *E. cava* (dieckol, 6,6′-bieckol, 8,8′-bieckol, eckol and phlorofucofuroeckol-A)	In vitro: assays of AChE, BChE and BACE-1 activities -- In vitro: Jurkat clone E1–6 cells (GSK3β activity at 50 μM)	Inhibition of AChE and BChE activities (IC_50_ 16.0–96.3 μM and 0.9–29.0 μM, respectively) Inhibition of BACE-1 activity (18.6–58.3% at 1 μM) Inhibition of GSK3β activity (14.4–39.7% at 50 μM)	[234]
Phlorotannin-rich extract from *E. bicyclis* (eckols)	In vitro: assays of AChE and BChE activities	Inhibition of AChE and BChE activities (IC_50_ 2.78 and 3.48 μg/mL, respectively)	[235]
Aqueous extracts of *Gracilaria beckeri*, *Gelidium pristoides*, *U. rigida and E. maxima* composed by phloroglucinol, catechin and epicatechin 3-glucoside	In vitro: assays of AChE and BChE activities	High antioxidant potency Inhibition of AChE and BChE activities (IC_50_ 49.41 and 52.11 μg/mL, respectively, for *E. maxima*) Inhibition of Aβ aggregation	[236]
Aqueous-ethanolic extracts from *E. maxima*, *G. pristoides*, *Gracilaria gracilis*, and *Ulva lactuca* containing phlorotannins, flavonoids and phenolic acids	In vitro: assays of AChE, BChE and BACE-1 activities	Inhibition of AChE and BChE activities (IC_50_ 1.74–2.42 and 1.55–2.04 mg/mL, respectively) Inhibition of BACE-1 activity (IC_50_ 0.052–0.062 mg/mL) Inhibition of Aβ aggregation	[237]
Phlorofucofuroeckol isolated from *E. cava*	In vitro: Glutamate-stimulated PC12 cells (10 μM of phenol)	Increased the cell viability and attenuated glutamate excitotoxicity Inhibited the apoptosis in a caspase-dependent manner Regulated the production of ROS and attenuated mitochondrial dysfunction	[238]
Phloroglucinol isolated from *E. cava*	In vitro: Aβ-induced neurotoxicity in HT-22 cells (10 μg/mL) --- In vivo: 5XFAD mice, model of AD (acute, 1.2 μmol of phenol bilaterally delivery)	Reduced the Aβ-induced ROS accumulation in HT-22 cells Ameliorated the reduction in dendritic spine density --- Attenuated the impairments in cognitive dysfunction	[239]
Eckmaxol from *E. maxima*	In vitro: Aβ oligomer-induced neurotoxicity in SH-SY5Y cells (5–20 μM of phenol)	Prevented the Aβ oligomer-induced neurotoxicity Inhibition of GSK3β and ERK signaling pathway	[240]
*E. cava* rich in phlorotannins (eckol, 8,80-bieckol and dieckol)	In vitro: Aβ 25–35-induced damage in PC12 Cells (1–50 μM of phenol)	Inhibition of pro-inflammatory enzymes preventing Aβ production and neurotoxicity on the brain	[241]
Phlorotannin-rich fraction from *Ishige foliacea*	In vivo: scopolamine-induced amnesic mice (50 and 100 mg/kg bw/day of extract orally administered for 6 weeks)	Inhibition of AChE activity in the brain Improved the status antioxidant Prevented the memory impairment via regulation of ERK–CREB–BDNF pathway	[242]

AD: alzheimer’s disease; AChE: acetylcholinesterase; BChE: butyrylcholinesterase; BACE-1: beta-site amyloid precursor protein cleaving enzyme 1; Aβ: amyloid-β peptides; GSK3β: glycogen synthase kinase 3β; ROS: reactive oxygen species; ERK: extracellular signal-regulated kinase; BDNF: brain-derived neurotrophic factor.

Glutamate is an important neurotransmitter responsible for memory, learning and cognitive function. However, excessive glutamate release from the presynaptic terminals has also been suggested as a mechanism for increased Aβ production via NMDA receptor-mediated Ca^2+^ influx [243]. Hence, administering biological active compounds capable of protecting the brain cells against glutamate excitotoxicity may be an appealing therapeutic intervention. Phlorofucofuroeckol isolated from *E. cava* increased cell viability in glutamate-stimulated PC12 cells, attenuating glutamate excitotoxicity. Kim et al. [238] showed that phlorofucofuroeckol inhibits glutamate-induced apoptotic cell death in a caspase-dependent manner, regulates the production of ROS and attenuates mitochondrial dysfunction.

No drug has been developed yet to combat Aβ aggregation, although some marine phenolics have shown the ability to attenuate Aβ-induced neurotoxicity in AD models. Phloroglucinol isolated from *E. cava* reduced ROS generation caused by Aβ-induced neurotoxicity in HT-22 cells. Yang et al. [239] have shown that phloroglucinol ameliorated the reduction in dendritic spine density induced by Aβ treatment in rat primary hippocampal neuron cultures. Administration of phloroglucinol to the hippocampal region attenuated the impairments in cognitive dysfunction 5XFAD mice, an animal model of AD [239]. Eckols from *E. cava* demonstrated it is able to inhibit glycogen synthase kinase 3β (GSK3β), which inhibits the biosynthesis of amyloid precursor proteins and is related to the formation of hyperphosphorylated tau and the generation of Aβ [234]. Likewise, eckmaxol isolated from *Ecklonia maxima* also prevented Aβ oligomer induced neurotoxicity in SH-SY5Y cells, via the inhibition of glycogen synthase kinase 3β (GSK3β) and the ERK signaling pathway [240]. Neurodegenerative disorders are often characterized by a wide range of diverse and intertwined neuro-inflammatory processes, leading to primary or secondary central nervous system damage. A recent study showed that eckols, 8,8’-bieckol and dieckol, were able to inhibit TNFα, IL-1β and prostaglandin E2 (PGE2) production at protein level, related to the down-regulation of proinflammatory enzymes, iNOS and COX-2, through the negative regulation of the NF-kB pathway in Aβ_25–35_-stimulated PC12 cells, preventing the neurotoxicity on the brain. Especially, dieckol showed strongest anti-inflammatory effects via suppression of p38, ERK and JNK [241]. Um et al. [242] assessed the neuroprotective activity of a phlorotannin-rich fraction from *Ishige foliacea* on mice with scopolamine-induced memory impairment. A supplementation of 50 and 100 mg/kg of the phlorotannin-rich fraction for 6 weeks improved the memory impairment symptoms of the rodents, reduced AChE activity in their brain and improved their antioxidant status by decreasing lipid peroxidation levels and increasing glutathione levels and SOD activity. Additionally, the phlorotannin-rich supplementation up-regulated the expression levels of: brain-derived neurotrophic factor (BDNF), tropomyosin receptor kinase B, phosphorylated ERK and cyclic AMP-response element-binding protein (CREB). Therefore, the phlorotannin-rich fraction prevented the memory impairment via regulation of the ERK–CREB–BDNF pathway.

In summary, marine phenolics show potential to prevent or delay the consequences of AD (Table 7 and Figure 2), although it is still a little explored pathology and more in vitro studies need to be undertaken. In addition, the blood–brain barrier represents a challenge for the bioavailability of these compounds, and although there are a few studies confirming that dietary polyphenols may cross the blood–brain barrier [244], it is necessary to confirm the results derived from in vitro models in in vivo studies.

### 3.5. Cancer

Cancer represents a group of diseases related to the abnormal proliferation of any of the different kinds of cells in the body with the potential to spread to other parts of the body [245]. The side effects of antineoplastic drugs and chemotherapy motivate the search for natural products that could be used as new therapeutic agents with more efficacy, specificity and without adverse effects. Among the bioactive compounds present in marine sources, polyphenols have been demonstrated to have potent anticancerogenic activity, which has been recently reviewed [36,246,247]. An association of dietary seaweed intake (gim, miyeok and dashima) with single-nucleotide polymorphisms (SNPs; rs6983267, rs7014346, and rs719725) and colorectal cancer risk in a Korean population has been established. 

Colorectal cancer risk and c-MYC rs6983267 association was derived from an analysis of 923 patients and 1846 controls [248]. Furthermore, an inverse association between dietary seaweed intake (gim, miyeok, and dashima) and colorectal cancer risk was observed, suggesting that dietary seaweed may have a positive benefit as a chemotherapeutic or chemopreventive agent for colorectal cancer risk associated with the rs6983267 genotype. Although phenolic compounds are not the only bioactives present in the consumed dietary seaweeds, it is well known that they can contribute to preventing or slowing down carcinogenic processes through the different mechanisms that are discussed next (Table 8 and Figure 2).

Polyphenol-rich extracts, as well as isolated phlorotannins and bromophenols, have been extensively described as inhibitors of cancer cell proliferation (Table 8). Aqueous extracts derived from brown *Cystoseira crinita* showed a significant antiproliferative activity against colon (HCT15) and breast (MCF7) human tumor cell lines, and these were associated with the total phenolic content and the antioxidant activity of the extracts [249]. Likewise, a phlorotannin-rich extract from *A. nodosum* inhibited the viability of colon carcinoma HT29 cells [250]. Montero et al. [127] evaluated five purified hydroalcoholic extracts of *S. muticum* from the North Atlantic coast. Their results revealed that the *S. muticum* sample with the highest level of total phlorotannins presented the highest antiproliferative activity against HT29 adenocarcinoma colon cancer cells (IC_50_~53–58 μg/mL after 24 h of treatment). Phlorotannins isolated from the brown alga *E. maxima* (phloroglucinol, eckol, 7-phloroeckol and 2-phloroeckol) showed antiproliferative activity in HeLa, H157 and MCF7 cancer cell lines, with eckol being the most bioactive tested phlorotannin (IC_50_ < 50 μg/mL against HeLa and MCF7 cells after 24 h of treatment) [251]. Namvar et al. [252] evaluated the antiproliferative activity against five human cancer cell lines (MCF-7, MDA-MB-231, HeLa, HepG2, and HT-29) of seaweed alcoholic extracts of red (*Gracillaria corticata*), green (*Ulva fasciata*) and brown (*Sargassum ilicifolium*). All the extracts showed a dose-dependent antiproliferative activity against all the cancer cell lines, although *G. corticata* had the greatest inhibition activity against MCF-7 cell line (IC_50_ value of 30 μg/mL after 24 of treatment). Lopes-Costa et al. [253] reported that phloroglucinol not only reduced the growth of two colorectal cancer cell lines (HCT116 and HT29), but also intensified the activity of 5-fluorouracil, one of the most commonly used chemotherapeutic drugs to treat colorectal cancer. Two polybrominated diphenyl ethers, 3,4,5-tribromo-2-(2’,4’-dibromophenoxy)-phenol and 3,5-dibromo-2-(2’,4’-dibromophe-noxy)-phenol, which were isolated from *Dysidea* sp., an Indonesian marine sponge, showed antiproliferative activity against PANC-1 cells under glucose-starved conditions. The first bromophenol might act by inhibiting complex II in the mitochondrial electron transport chain [254].

There are many studies focusing on the isolation of seaweed extracts rich in bioactive compounds, along with their chemical characterization and antiproliferative activity. Zenthoefer et al. [24] determined the cytotoxic potential of different extracts from *F. vesiculosus* L. against human pancreatic cancer cells (Panc89 and PancTU1) and the most active extract (IC_50_ value of 72 μg/mL against Panc89 and 77 μg/mL against PancTU1 cells after 72 h of treatment) was characterized by H-1—NMR spectroscopy, identifying two chemical structures belonging to the phlorotannin group. Bernardini et al. [255] explored the chemical composition of French Polynesian *P. pavonica* extract by spectrophotometric assays (total phenolic compounds, tannin content and antioxidant activity) and GC–MS analysis to obtain extracts with improved antiproliferative and pro-apoptotic activities against two osteosarcoma cell lines, SaOS-2 and MNNG. Likewise, extracts of three brown marine macroalgae *Dictyota dichotoma*, *P. pavonica* and *Sargassum vulgare* were tested for improving their antioxidant, antimicrobial and cytotoxic activities on human colon carcinoma LS174 cells, human lung carcinoma A549 cells, malignant melanoma FemX cells and chronic myelogenous leukemia K562 cells [256]. Sevimli-Gur and Yesil-Celiktas [155] extracted detached leaves of *Posidonia oceanica* and *Zostera marina* with CO_2_, with ethanol as co-solvent, to obtain phenolic acids with cytotoxic properties on breast, cervix, colon, prostate and neuroblastoma tumor cells. *Z. marina* extract showed the best IC_50_ values of 25, 20 and 8 μg/mL after 48 h in neuroblastoma, colon and cervix cancer cell lines, respectively. In the same line, optimized extraction, preliminary chemical characterization, and evaluation of the in vitro antiproliferative activity of phlorotannin-rich fraction from brown seaweed, *Cystoseira sedoides*, was developed by Abdelhamid et al. with promising results (IC_50_ value of 78 μg/mL after 72 h of treatment) [257]. Another recent example was carried out by Abu-Khudir et al. [258], who evaluated the antioxidant, antimicrobial and cytotoxic effect of crude extracts of the Egyptian brown seaweeds, *Sargassum linearifolium* and *Cystoseira crinita*, against a group of cancer cells—the latter with a strong cytotoxic activity against MCF-7 cells (IC_50_ value of 18 μg/mL after 48 h). They observed an increased mRNA and protein expression of the pro-apoptotic Bax and the marker of autophagy Beclin-1, a reduced expression of the anti-apoptotic Bcl-2, as well as revealed the ability of these extracts to induce apoptosis and autophagy in MCF-7 cells. Finally, Premarathana et al. [259] carried out a preliminary screening of the cytoxicity activity on a mouse fibroblast (L929) cell line of twenty-three different seaweed species in Sri Lanka (Table 8). Crude extracts of brown and red seaweed species showed high mortality rate compared to green seaweeds and *Jania adherens* showed a remarkable cytotoxic effect on L929 cell line (51% cell viability compared with control after 24 h). 

Activation of apoptosis, programmed cell death, is an important target in cancer therapy. Namvar et al. [252] demonstrated the ability of an alcoholic extract from the red seaweed *Gracillaria corticata* to induce apoptosis in human breast cancer cells (MCF-7), as well as *Sargassum linearifolium* and *Cystoseira crinite*, as already mentioned [258]. Dieckol suppressed ovarian cancer cell (SKOV3) growth by inducing caspase-dependent apoptosis via ROS production and the regulation of Akt and p38 signaling pathways [260]. A phlorotannin-rich extract from *E. cava*, mainly composed of dieckol, was assessed in terms of cisplatin responsiveness, and in its effects on A2780 and SKOV3 ovarian cancer cell lines, as well as on a SKOV3-bearing mouse model [261]. They found that dieckol may improve the efficacy of platinum drugs for ovarian cancer, by enhancing cancer cell apoptosis via the ROS/Akt/NFκB pathway and reducing nephrotoxicity. Phlorofucofuroeckol A, a phlorotannin present in the brown alga *E. bicyclis*, exhibited antiproliferative and proapoptotic properties in human cancer cells (LoVo, HT-29, SW480 and HCT116) by activating the transcription factor 3 (ATF3)-mediated pathway in human colorectal cancer cells [262]. Park et al. [263] showed that an ethanolic extract of *Hizikia fusiforme* decreased the viability of B16F10 mouse melanoma cells and induced apoptosis through activation of extrinsic and intrinsic apoptotic pathways and ROS-dependent inhibition of the PI3K/Akt signaling pathway. No chemical characterization of the tested extract was carried out, and other bioactive compounds present in the extract, apart from polyphenols, could have contributed to the observed effect.

Metastasis is an important cellular marker of cancer progression and has been associated with an increase in the activity of matrix metalloproteinases (MMPs), which are needed to degrade connective tissues. A polyphenol-rich extract of *E. cava* showed a potent inhibitory effect on the metastatic activity of A549 human lung carcinoma cells, including the suppressions of migration and invasion. This polyphenol-rich extract down-regulated MMP-2 activity through the inhibition of the PI3K/Akt signaling pathway [264]. Phloroglucinol, isolated from the brown alga *E. cava*, diminished the population of breast cancer cell lines (MCF7, SKBR3 and BT549) in tumors, by inhibiting KRAS and its downstream PI3K/Akt and RAF-1/ERK signaling pathways. Furthermore, phloroglucinol increased sensitization of breast cancer cells to conventional therapy (chemotherapy and ionizing radiation) [265]. The same research group also confirmed the effectiveness of phloroglucinol against metastasis of breast cancer through downregulation of SLUG by the inhibition of PI3K/Akt and RAS/RAF-1/ERK signaling pathways [266]. Phloroglucinol was also effective against metastasis of breast cancer cells, drastically suppressing their metastatic ability in lungs, and extending the survival time of mice. In agreement with in vitro data, phloroglucinol also exhibited breast anticancer activity at 25 mg/kg bw, either by decreasing tumor growth or by suppressing the metastatic ability of breast cancer cells that spread to the lungs, contributing in both cases to an increase in survival time in mice [266].

Angiogenesis has a crucial role in tumor growth and metastasis and is also related to an aggressive tumor phenotype where vascular endothelial growth factor (VEGF) is the most important component. Qi et al. [267] demonstrated that bis(2,3-dibromo-4,5-dihydroxybenzyl) ether treatment repressed angiogenesis in human endothelial cells (HUVECs) and in zebrafish embryos via inhibiting the VEGF signal systems. Dieckol modulated the expression of key molecules that regulate apoptosis, inflammation, invasion, and angiogenesis. Daily administration of dieckol isolated from *E. cava* (40 mg/kg for 15 weeks) to rats with N-nitrosodiethylamine(NDEA)-induced hepatogenesis regulated xenobiotic-metabolizing enzymes and by modulating Bcl-2 family proteins induced apoptosis via the regulation of mitochondrial release of cytochrome c and the activation of caspases [268]. Anti-inflammatory activity of dieckol was associated with inhibition of the nuclear factor-kappa B (NF-κB) and COX2. In addition, dieckol treatment inhibited invasion by decreasing proliferating cell nuclear antigen (PCNA) expression and angiogenesis by changing MMP-2 and MMP-9 activities and VEGF expression. Li et al. [269] found that dieckol exhibited antiangiogenic activity by inhibiting the proliferation and migration of EA.hy926 cells through mitogen-activated protein kinase (MAPK), extra-cellular signal regulated kinase (ERK) and p38 signaling pathways (Table 8).

The antioxidant activity of phlorotannins and bromophenols offers a complementary mechanism to mitigate cancerous processes as observed in a few studies already discussed. Zhen et al. [270] associated the protective effects of eckol against PM2.5-induced cell damage on human HaCaT keratinocytes with a reduced ROS generation, ensuring the stability of molecules, and maintaining a steady mitochondrial state. In addition, eckol protected cells from apoptosis by inhibiting the MAPK signaling pathway. An interesting study carried out by Zhang et al. [271] investigated the in vivo antitumor effect and the mechanisms involved in a sarcoma 180 (S180) xenograft-bearing animal model supplemented with low-dose (0.25 mg/kg), middle-dose (0.5 mg/kg) and high-dose (1.0 mg/kg) of eckol. The pro-apoptosis and antiproliferation activities of eckol were manifested by the increased TUNEL-positive apoptotic cells, the upregulated Caspase-3 and Caspase-9 expression, and the downregulated expression of Bcl-2, Bax, EGFR and p-EGFR in eckol-treated transplanted S180 tumors. Eckol stimulated the mononuclear phagocytic system, recruited and activated DCs, promoted the tumor-specific Th1 responses, increased the CD4+/CD8+ T lymphocyte ratio, and enhanced cytotoxic T lymphocyte responses in the eckol-treated animals; this suggests its potent stimulatory property on innate and adaptive immune responses.

Despite the promising anticancer activity described for marine phenolics, no human studies have been conducted to directly confirm their efficacy against cancer. DNA damage results in an increased rate of genetic mutations that often lead to the development of cancer [272]. The anticancerogenic activity of seaweeds was indirectly verified in a clinical trial. A modest improvement in DNA damage was observed in an obese group after consuming 100 mg/day for 8 weeks of a (poly)phenol-rich extract of the brown algae *A. nodosum*.

In summary, there are many in vitro studies—in addition to in vivo studies—using animal models that demonstrate the potential of marine polyphenols to block carcinogenic mechanisms (Table 8 and Figure 2). Given the prevalence of this pathology, the next step would be to test their efficacy in human trials.

**Table 8:** Effect of marine phenolics on the prevention of cancer.

Compounds/Marine Source	Test Model	Outcome	Ref.
Dietary seaweed intake (gim, miyeok, and dashima)	In vivo: 923 colorectal cancer patients and 1846 controls	Association between c-MYC rs6983267 and colorectal cancer risk Inverse association between dietary seaweed intake and colorectal cancer risk	[248]
Aqueous extract derived from brown *Cystoseira crinita*	In vitro: HCT15 and MCF7 cells (25–250 μg/mL for extracts)	Antiproliferative activity (IC_50_ of 10.5–26.4 μg/mL on HCT15 and 17.9–29.5 μg/mL for 24 h) associated with phenolic content and antioxidant activity	[249]
Phlorotannin-rich extract from *A. nodosum*	In vitro: HT29 cells (100–500 μg/mL for extracts)	Antiproliferative activity	[250]
Ethanolic extract from *S. muticum* rich in phlorotannins	In vitro: HT29 cells (12.5–100 μg/mL for extracts)	Antiproliferative activity (IC_50_ of ~53.5–57.9, 55.0–57.8 and 59.4–74.0 μg/L for 24, 48 and 72 h of treatment of *S. muticum* extracts)	[127]
Phlorotannins isolated from *Ecklonia maxima* (phloroglucinol, eckol, 7-phloroeckol and 2-phloroeckol)	In vitro: HeLa, H157 and MCF7 cells (6.25–500 μg/mL for phenol)	Antiproliferative activity: eckol was the most active of all the tested phlorotannins against HeLa and MCF7 cells after 24 of treatment (IC_50_ < 50 μg/mL)	[251]
Alcoholic extract from red (*Gracillaria corticata*), green (*Ulva fasciata*) and brown (*Sargassum ilicifolium*) seaweeds	In vitro: MCF-7, MDA-MB-231, HeLa, HepG2 and HT-29 cells (15–300 μg/mL for extracts)	Antiproliferative activity: *G. corticata* extract had the greatest activity against MCF-7 cells (IC_50_ of 30, 37, 53, 102 and 250 μg/mL on MCF-7, HeLa, MDA-MB-231, HepG2 and HT-29 cells, respectively, after 24 h of treatment) *G. corticata* extract induced the apoptosis in human breast cancer cells	[252]
Phloroglucinol	In vitro: HCT116 and HT29 cells (10–300 μM of phenol)	Antiproliferative activity Intensified the 5-fluorouracil activity	[253]
3,4,5-Tribromo-2-(2’,4’-dibromophenoxy)-phenol (**1**) and 3,5-dibromo-2-(2’,4’-dibromophenoxy)-phenol (**2**) isolated from marine sponge *Dysidea* sp.	In vitro: PANC-1 cells under glucose-starved conditions (1–100 μM of phenol)	Antiproliferative activity (IC_50_ values of 2.1 and 3.8 μM for 1 and 2, respectively, after 12 h) Inhibition of the complex II in the mitochondrial electron transport chain	[254]
Different extracts from *F. vesiculosus L.* rich in phlorotannins	In vitro: Panc89 and PancTU1 cells (0.8–500 μg/mL for crude extracts and 0.16–200 μg/mL for fractions)	Antiproliferative activity (IC_50_ of 72 μg/mL against Panc89 and of 77 μg/mL against PancTU1 cells after 72 h of treatment for the most active crude extract)	[24]
Extract from *P. pavonica*	In vitro: SaOS-2 and MNNG cells (0.5–2.5 μg/mL for extract)	Antiproliferative (IC_50_ value of 152.2 and 87.75 μg/mL for SaOS-2 and MNNG cells, respectively, after 24 h) and pro-apoptotic activities	[255]
Extracts of three brown marine macroalgae *Dictyota dichotoma*, *Padaina pavonia* and *Sargassum vulgare*	In vitro: LS174, A549, FemX, K562 cells (12.5–200 μg/mL for extract)	Characterization of the cytotoxic activity *D. dichotoma* showed the strongest cytotoxic activity of all the tested extracts (IC_50_ values ranging from 9.76 to 50.96 μg/mL after 72 h)	[256]
Extracts from detached leaves *of Posidonia oceanica* and *Zostera marina*	In vitro: MCF-7, MDA-MB-231, SK-BR-3, HT-29, HeLa, PC-3 and Neuro 2A cells, as well as African green monkey kidney (VERO) (6.25–100 μg/mL for extract)	Characterization of the cytotoxic activity *Z. marina* extract showed the best IC_50_ values of 25, 20 and 8 μg/mL after 48 h in neuroblastoma, colon and cervix cancer cell lines, respectively	[155]
Phlorotannin-rich fraction from *Cystoseira sedoides*	In vitro: MCF-7 cells (10–200 μg/mL for extract)	Characterization of the antiproliferative activity (IC_50_ value of 78 μg/mL after 72 h)	[257]
Crude extracts from two Egyptian brown seaweeds, *Sargassum linearifolium* and *Cystoseira crinita*	In vitro: a panel of cancer cells such as MCF-7 cells, among others (0.01–2000 μg/mL for extract)	Characterization of the cytotoxic activity. *C. crinite* cold methanolic extract showed a strong cytotoxic activity against MCF-7 cells (IC_50_ value of 18 μg/mL after 48 h) Induced the apoptosis and autophagy in MCF-7 cells	[258]
Aqueous seaweed extracts of 23 different species in Sri Lanka	In vitro: L929 cells (10–100 μg/mL for extract)	Antiproliferative activity Crude extracts of brown and red seaweeds species have shown high mortality rate compared to green seaweeds *Jania adherens* showed a remarkable cytotoxic effect on L929 cell line (51% cell viability compared with control after 24 h)	[259]
Ethanolic extract from *E. cava* whose main component was dieckol	In vitro: A2780 and SKOV3 cells	Cytotoxic effects on A2780 and SKOV3 ovarian cancer cells (IC_50_ ranging from 84 to 100 μg/mL for extract and from 77 to 169 μM for phenols, with dieckol being the most active of all, after 24 h) Induced the apoptosis on SKOV3 cells via Akt and p38 signaling pathways	[260]
Phlorotannin-rich extract from *E. cava* rich in dieckol	In vitro: A2780 and SKOV3 cells (50–100 μg/mL) In vivo: SKOV3-bearing mouse model (75 and 150 mg/kg bw for extract and 50 and 100 mg/kg bw for dieckol was given orally three times/week for 4 weeks)	Phlorotannin-rich extract may improve the efficacy of cisplatin for ovarian cancer by enhancing cancer cell apoptosis via the ROS/Akt/NFkB pathway	[261]
Phlorofucofuroeckol A present in *E. bicyclis*	In vitro: LoVo, HT-29, SW480 and HCT116 cells (25–100 μM of phenol)	Antiproliferative and pro-apoptotic properties Induced the apoptosis on colorectal cancer cells by ATF3 signaling pathway	[262]
Ethanolic extract of *H. fusiforme*	In vitro: B16F10 cells (25–400 μg/mL of extract)	Cytotoxic activity Induced the apoptosis through activation of extrinsic and intrinsic apoptotic pathways and ROS-dependent inhibition of the PI3K/Akt signaling pathway	[263]
Phlorotannin-rich extract from *E. cava* rich in phenolic compounds	In vitro: A549 cells (12.5–50 μg/mL of extract)	Inhibition of metastatic activity including suppression of migration and invasion Down-regulated the MMP-2 activity via PI3K/Akt	[264]
Phloroglucinol isolated from *E. cava*	In vitro: MCF7, SKBR3 and BT549 cells (10–100 μM of phenol) In vivo: MDA-MB231 breast cancer cells implanted into mammary fat pads of NOD-scid gamma (NSG) mice, treated with phloroglucinol 4 times on alternate days (25 mg/kg bw by intratumoral injections)	Antiproliferative effect by KRAS inhibition and its downstream PI3K/Akt and RAF-1/ERK signaling pathways Increased the sensitization of breast cancer cells to conventional therapy	[265]
Phloroglucinol isolated from *E. cava*	In vitro: BT549 and MDA-MB-231 cells (10–100 μM of phenol) In vivo: GFP-labeled metastatic MDA-MB231 cells transplanted into mammary fat pads of NSG mice, treated with phloroglucinol 4 times on alternate days (25 mg/kg bw by intraperitoneal injection)	Inhibited the metastatic ability of breast cancer cells Decreased the expression of SLUG, EMT master regulator through inhibition of PI3K⁄Akt and Ras⁄Raf-1 ⁄ERK Inhibited the in vivo metastatic ability of breast cancer cells	[266]
Bis(2,3-dibromo-4,5-dihydroxybenzyl) ether	In vitro: HUVEC cells (12.5–50 μM of phenol) In vivo: Zebrafish embryos model (6.25–25 μM of phenol)	Repressed the angiogenesis in both in vitro and in vivo models by inhibiting the VEGF signal systems	[267]
Dieckol from *E. cava*	In vivo: *N*-nitrosodiethylamime-induced hepatocarcinogenesis rats (40 mg/kg bw/day for 15 weeks administered orally)	Regulated the xenobiotic-metabolizing enzymes Induced the apoptosis by mitochondrial pathway Inhibited the invasion by decreasing PCNA expression Inhibited the angiogenesis by changing MMP-2 and MMP-9 activity and VEGF expression Anti-inflammatory activity by inhibiting NF-kB and COX2	[268]
Dieckol	In vitro: EA.hy926 cells (10–100 μM of phenol)	Antiangiogenic activity by inhibiting the proliferation and migration of cells through MAPK, ERK and p38 signaling pathways	[269]
Eckol	In vitro: on human HaCaT keratinocytes against PM2.5-induced cell damage (30 μM of phenol for 17 days)	Decreased the ROS generation Protected the cells from apoptosis by inhibiting MAPK signaling pathway	[270]
Eckol	In vivo: sarcoma 180 (S180) xenograft-bearing animal model supplemented with low dose (0.25 mg/kg bw), middle dose (0.5 mg/kg bw) and high dose (1.0 mg/kg bw) of phenol administered orally	Proapoptotic and antiproliferative activities by improving the immune response	[271]
Polyphenol-rich extract from *A. nodosum*	In vivo: 80 overweight or obese population (100 mg/day of extract for 8 weeks)	Improvements in DNA damage in the obese subset	[30]

ROS:reactive oxygen species; Akt: protein kinase B; NFkB: nuclear factor kappa B; ATF3: transcription factor 3; MMP: metalloproteinase; PI3k: phosphoinositide 3-kinase; VEGF: vascular endothelial growth factor; PCNA: proliferating cell nuclear antigen; COX-2: cyclooxygenase-2; MAPK: mitogen-activated protein kinase; ERK: extracellular signal-regulated kinase; DNA: deoxyribonucleic acid.

### 3.6. Human Gut Microbiota

The human intestine contains an intricate ecological community of dwelling bacteria, referred to as gut microbiota, which plays a pivotal role in host homeostasis. Multiple factors could interfere with this delicate balance, including genetics, age, antibiotics, as well as environmental factors, particularly diet, thus causing a disruption of microbiota equilibrium (dysbiosis). Growing evidence supports the involvement of gut microbiota dysbiosis in gastrointestinal and extra-intestinal cardiometabolic diseases, namely obesity and diabetes [273]. Even though, seaweeds and microalgae are excellent sources of prebiotics such as fucoidans, alginates, carrageenans and exopolysaccharides that can be partially fermented. We will focus next on marine polyphenol studies that explore their influence on gut microbiota (Table 9).

**Table 9:** Effect of marine phenolics in human gut microbiota.

Compounds/Marine Source	Test Model	Outcome	Ref.
Food supplement from *Kappaphycus alvarezii*	In vivo: rats fed for 8 weeks on high-carbohydrate, high-fat diet, alone or supplemented with 5% (*w/w*) algae	Improved the cardiovascular, liver and metabolic biomarkers in obese rats Modulated the balance between *Firmicutes* and *Bacteroidetes* in the gut	[226]
Polyphenol-rich extracts from brown macroalgae *L. trabeculate*	In vivo: high-fat diet and STZ-induced diabetic rats (200 mg/kg/day bw of phenol for 4 weeks by gavage)	Attenuated the hyperglycemia in diabetic rats Increased the short-chain fatty acid contents in fecal samples Enhanced the abundance of *Bacteroidetes*, *Odoribacter* and *Muribaculum* Decreased the abundance of *Proteobacteria* as well as *Firmicutes/Bacteroidetes* ratio	[103]
Water-ethanolic extract of green macroalgae *Enteromorpha prolifera* rich in flavonoids	In vivo: STZ-induced diabetic rats (150 mg/kg/day bw of phenol for 4 weeks by gavage)	Showed the antidiabetic activity on diabetic mice Modulated the balance between *Firmicutes* and *Bacteroidetes* in the gut and increased the abundance of the *Lachnospiraceae* and *Alisties* bacteria involved in the prevention of T2DM	[191]
Water-soluble compounds from *Nitzschia laevis* extract	In vivo: high-fat diet obese mice (50 mg/kg/day bw of extract for 8 weeks by gavage)	Prevented obesity in mice Protected the gut epithelium and positively reshaped the gut microbiota	[274]

Several studies showed that polyphenol-rich extracts had a positive effect on regulating the dysbiosis of the microbial ecology in rats. The red seaweed *K. alvarezii* tested as a food supplement demonstrated its capacity to improve cardiovascular, liver, and metabolic biomarkers in obese rats. *Kappaphycus* also modulated the balance between *Firmicutes* and *Bacteroidetes* in the gut, which could serve as a potential mechanism to reverse MetS through selective inhibition of obesogenic gut bacteria and promote healthy gut bacteria [226]. Polyphenol-rich extracts from *L. trabeculate* attenuated hyperglycemia in high-fat diet and STZ-induced diabetic rats [103], as aforementioned. Higher *Bacteroidetes*, *Odoribacter* and *Muribaculum* abundances, lower *Proteobacteria* abundances, as well as a reduced *Firmicutes/Bacteroidetes* ratio, were observed in the polyphenol supplemented group in comparison with untreated diabetic rats. In addition, rats supplemented with polyphenols showed higher amounts of short-chain fatty acids in fecal samples compared with the un-supplemented diabetic group. In their study discussed above, Yan et al. [191] showed the antidiabetic activity of a water-ethanolic extract of the green macroalgae *E. prolifera.* This extract, which was rich in flavonoids, significantly modulated the balance between *Firmicutes* and *Bacteroidetes* and increased the abundance of the *Lachnospiraceae* and *Alisties* bacteria involved in the prevention of T2DM. Guo et al. [274] demonstrated the efficacy of administering 50 mg/kg/day for 8 weeks of a *Nitzschia laevis* extract in preventing obesity in mice fed with a high-fat diet. This extract protected the gut epithelium and positively reshaped the gut microbiota composition against the damaging effect of a high-fat diet. The *Nitzschia laevis* extract was a mixture of bioactive compounds, including carotenoids and polyphenols; therefore, the specific functional ingredient(s) of this product and their potential synergistic effect (if any) are yet to be defined.

### 3.7. Infectious Diseases

Apart from the dietary and lifestyle-related diseases, marine phenolics are involved in the prevention of other pathological processes due to their multiple bioactivities (enzyme inhibitory effect and antimicrobial, antiviral, anticancer, antidiabetic, antioxidant, and anti-inflammatory activities, among others). Special attention should be focused on infectious diseases caused by bacteria, viruses, and fungi that continue to grow despite the development of antibiotics in the 1940s. In the western world, the issue is not the availability of antimicrobial treatments, but the developed immunity of microorganisms to pharmaceutical drugs and disinfectants. Natural products are an important source of new drugs. Approximately 80 antibacterial drugs, which were approved from 1981 to 2014, either were natural products or directly derived from them [275]. Therefore, bacterial and fungal infections and the emerging multidrug resistance are driving interest into fighting these microorganisms with natural products, which have generally been considered complementary to pharmacological therapies, and marine phenolics can be an appealing alternative (Table 10).

Lopes et al. [131] found that in vitro phlorotannin purified extracts from ten brown algal species, collected along the Portuguese west coast, were shown to be less effective against fungi and Gram-negative bacteria than Gram-positive bacteria. *F. spiralis* and *C. nodicaulis* were the most effective species (MIC = 3.9 mg/mL), followed by *C. usneoides*, *S. vulgare* (MIC = 7.8 mg/mL), and *C. tamariscifolia* (MIC = 31.3 mg/mL) against *Trichophyton rubrum*. Likewise, *C. nodicaulis* extracts were the most effective against *C. albicans* (MIC = 7.8 mg/mL). *Cystoseira* sp, and *F. spiralis* were the most active against *Staphylococcus* and against *M. luteus* (with minimum inhibitory concentration (MIC) values of 2.0–3.9 mg/mL). These effects could be related to their content in phlorotannins of the purified extracts, although their microbial activity is not truly relevant considering the MIC values. Rajauria et al. [276] reported that aqueous methanolic extracts isolated from the Irish brown seaweed *H. elongata* showed the highest antimicrobial activity against the Gram-positive bacteria *L. monocytogenes* and *E. faecalis*, and against the Gram-negative *P. aeruginosa* and *S. abony.* These authors related the antimicrobial activity with their polyphenol content and antioxidant activity.

Steele et al. [83] reported a “pseudo-induction” of plant phenolic acids (p-hydroxybenzoic acid, p-coumaric acid and vanillin) caused by changing the pattern of rearrangements of resources in plant tissues as a response of turtlegrass *Thalassia testudinum* to infection with *Labyrinthula* sp. The eelgrass *Zostera marina* possesses defensive mechanisms possibly associated with surface metabolites for surface protection and fouling control against marine epiphytic yeasts. The major constituents of eelgrass leaf surfaces and whole tissues were rosmarinic acid, p-coumaric acid, caffeic acid, ferulic acid, zosteric acid, apigenin-7-sulfate, luteolin-7-sulfate, diosmetin-7-sulfate (the most abundant) and their desulfated forms, as well as kaempferol-7,4′-dimethylether-3-O-sulfate. Papazian et al. [5] confirmed the existence of a selective chemical defense system in eelgrass which involved surface-associated phenolics and fatty acids to control growth and settlement of the microfouling yeasts *Cryptococcus fonsecae* and *Debaryomyces hansenii*. In addition, the antioxidant and cytotoxic capacities of desulfated flavonoids were enhanced compared to their sulfated compounds [5].

Free phenolic acid extracts from *Nannochloropsis* sp. (chlorogenic, gallic, protocatechuic, hydroxybenzoic, syringic, vanillic and ferulic acids) and *Spirulina* sp. (chlorogenic, hydroxybenzoic, protocatechuic and gallic acids) were efficient in reducing the mycelial growth rates of *Fusarium*. Moreover, synthetic mixtures of phenolic acids from both microalgae were less efficient than the natural extracts (EC_50_ values of 49.6 μg/mL and 33.9 μg/mL for *Nannochloropsis* and *Spirulina* phenolic acid extracts, respectively) to inhibit fungal growth, indicating that no purification is required [27]. Maadame et al. [50] evaluated the antimicrobial activities of nine marine microalgae from Moroccan coastlines (*Nannochloropsis gaditana*, *Dunaliella salina*, *Dunaliella* sp., *Phaeodactylum tricornutum*, *Isochrysis* sp., *Navicula* sp., *Chaeotoceros* sp., *Chlorella* sp. and *Tetraselmis* sp.). Ethanolic extracts of the selected microalgae were evaluated against bacteria (*Escherichia coli*, *Pseudomonas aeruginosa* and *Staphylococcus aureus*), yeast (*Candida albicans*) and fungus (*Aspergillus niger*). *Tetraselmis* sp. and *Nannochloropsis gaditana* extracts exhibited an inhibitory effect against the three types of bacteria while extracts from *Dunaliella salina*, *Phaeodactylum tricornutum* and *Isochrysis* sp. showed inhibitory activity only against the first two strains. *Tetraselmis* sp. was the most active of all the marine microalgae tested with MIC of 2.6 to 3.0 μg/mL of extract, indicative of high antimicrobial activity. All the tested extracts modestly inhibited the growth of *Candida albicans*, although *N. gaditana* showed the highest activity with MIC of 4.0 mg/mL of extract. None of them were able to inhibit *Aspergillus niger*. The observed antimicrobial activities were linked to fatty acid, carotenoid, and phenolic content of the extracts.

Sushanth and Rajashekhar [59] found that the extracts of four marine microalgae (*Chaetoceros calcitrans*, *Skeletonema costatum*, *Chroococcus turgidus* and *Nannochloropsis oceanica*) possessed effective inhibitory activity against *Staphylococcus aureus*, *Streptococcus pyogenes* and *Bacillus subtilis*. A hexane extract of *Chroococcus turgidus* showed significant inhibition activity against *Escherichia coli*, followed by an ethanol extract of *Skeletonema costatum* against *Streptococcus pyogenes.* Antifungal activity was found only in *Skeletonema costatum* and *Chroococcus turgidus* (Table 10).

Recently, Besednova et al. [277] have reviewed the activity of marine algal metabolites as promising therapeutics for the prevention and treatment of human immunodeficiency virus infection and acquired immunedeficiency syndrome (HIV/AIDS), discussing some studies focused on phlorotannins. Diphlorethohydroxycarmalol isolated from *Ishige okamurae* exhibited inhibitory effects on HIV-1 reverse transcriptase (RT) and integrase (IC_50_ values of 9.1 μM and 25.2 μM, respectively), although it did not show an inhibitory activity against HIV-1 protease [278]. Specifically, 6,6′-bieckol isolated from *E. cava* showed a strong inhibition against HIV-1 induced syncytia formation, lytic effects and viral p24 antigen production [279]. In addition, 6,6′-bieckol selectively inhibited the activity of HIV-1 RT enzyme and HIV-1 entry. Another compound of this group, 8,4′’-dieckol isolated from *E. cava* [280], also showed similar results as those reported by Artan et al. [279]. Therefore, there is enough evidence to support the antimicrobial activity of marine phenolics, which encourages the research community to continue exploring their application through the development of animal and human studies.

**Table 10:** Effect of marine phenolics on the prevention of infectious diseases.

Compounds/Marine Source	Test Model	Outcome	Ref.
Phlorotannins purified extracts isolated from ten brown algal species (*Cystoseira tamariscifolia*, *C. nodicaulis*, *C. usneoides*, *Sargassum vulgare*, *F. spiralis*, *Halopteris filicina*, *Stypocaulon scoparium*, *Cladostephus spongiosus*, *P. pavonica and Saccorhiza polyschides*) from Portugal	In vitro broth microdilution assay	Less effective against fungi than bacteria Phlorotannin extracts were more effective against Gram-positive than Gram-negative bacteria *Cystoseira* species and *F. spiralis* were the most active against *Staphylococcus* and *M. luteus* (minimum MIC of 2.0 mg/mL) *F. spiralis* and *C. nodicaulis* extracts were the most effective against the studied fungi (MIC = 3.9 mg/mL)	[131]
Aqueous methanolic extracts isolated from Irish brown seaweed *H. elongata*	In vitro broth microdilution assay	High antimicrobial activity against the Gram-positive bacteria, *L. monocytogenes* and *E. faecalis* High antimicrobial activity against the Gram-negative bacteria, *P. aeruginosa and S. abony*	[276]
Turtlegrass *Thalassia testudinum*	Inoculations of healthy turtlegrass blades with *Labyrinthula* sp.	The emergence of *Labyrinthula* sp. lesions on turtlegrass blades causes a “pseudo-induction” of plant phenolic acids as carbon resources over-accumulate in tissues located above wound sites	[83]
Extracts isolated from Eelgrass *Zostera marina*, whose leaf surface contained hydroxycinnamic acids, flavones and flavanols	In vitro bioassays against microbial foulers	Involvement of surface-associated phenolic compounds to control yeasts	[5]
Free phenolic acid extracts from *Nannochloropsis* sp. and *Spirulina* sp., as well as pure compounds	In vitro antifungal activity of phenols	Antifungal activity of phenolic acid extracts of the microalgae Higher activity of the natural free phenolic acid extracts (EC_50_ values of 49.6 μg/mL and 33.9 μg/mL for *Nannochloropsis* sp. and *Spirulina* sp., respectively) than the synthetic mixtures	[27]
Ethanolic extracts isolated from nine marine microalgae (*Nannochloropsis gaditana*, *Dunaliella salina*, *Dunaliella* sp., *Phaedactylum tricornutum*, *Isochrysis* sp., *Navicula* sp., *Chaeotoceros* sp., *Chlorella* sp. and *Tetraselmis* sp.)	In vitro broth microdilution assay	Variable inhibitory activity against *Escherichia coli*, *Pseudomonas aeruginosa* and *Staphylococcus aureus* (*Tetraselmis* sp. was the most active of all those tested with MIC of 2.6 to 3.0 mg/mL of extract) Inhibition of the growth of *Candida albicans* (*N. gaditana* showed the highest activity with a MIC of 4.0 mg/mL of extract) *Aspergillus niger* (fungus) was resistant to the effects of the extracts Activity of the extracts was due to the presence of fatty acids, carotenoids and phenols	[50]
Methanol, ethanol and hexane extracts from four marine microalgae (*Chaetoceros calcitrans*, *Skeletonema costatum*, *Chroococcus turgidus* and *Nannochloropsis oceanica*)	In vitro disc diffusion method	Inhibitory activity against *Staphylococcus aureus*, *Streptococcus pyogenes* and *Bacillus subtilis* Antifungal activity only in *Skeletonema costatum* and *Chroococcus turgidus*	[59]
Diphlorethohydroxycarmalol isolated from *Ishige okamurae*	In vitro antiviral enzyme assay	Inhibited the activity of HIV-1 reverse transcriptase and integrase with IC_50_ values of 9.1 μM and 25.2 μM, respectively	[278]
8,4′’-Dieckol isolated from *E. cava*	In vitro: H9, H9/HIV-1IIIB, CEM-SS, C8166 cells (1–50 μM of phenol)	Inhibited the activity of HIV-1 reverse transcriptase (RT) enzyme (91% inhibition ratio at 50 μM) and HIV-1 entry Exhibited the inhibitory effects against HIV-1 induced syncytia formation, lytic effects and viral p24 antigen production	[280]
6,6′-Bieckol isolated from *E. cava*	In vitro: H9, H9/HIV-1IIIB, CEM-SS, C8166 cells (0.1–30 μM of phenol)	Inhibited the activity of HIV-1 RT enzyme (EC_50_ 1.07 μM) as well as HIV-1 entry Exhibited the inhibitory effects against HIV-1 induced syncytia formation (EC_50_ 1.72 μM), lytic effects (EC_50_ 1.23 μM) and viral p24 antigen production (EC_50_ 1.26 μM)	[279]

MIC: minimum inhibitory concentration; HIV-1: human immunodeficiency virus-1; RT: reverse transcriptase; AIDS: acquired immunedeficiency syndrome.

## 4. Conclusions

Marine organisms represent a widely available and renewable source of bioactives, many of them found exclusively in this environment. Phenolics are among the most active families, but contrarily to those found in terrestrial sources, marine phenolics are much less studied. Advances in the analysis of their complex and diverse structure are desirable. These tools allow their characterization, needed both for commercialization and for the study of the structure activity relationships. Classical reverse phase (RP) chromatography is the most used approach but slightly ineffective since the hydrophobic stationary phase of RP columns weakly retain these compounds that, in addition to the close polar nature among the extensively isomerized phlorotannins, make their right resolution difficult. Thus, MS^n^ coupled to chromatographic techniques is widely used based on their mass-to-charge ratio (*m/z*) and fragmentation patterns (*m/z* of precursor and product ions, respectively). Quadrupole time-of-flight (qTOF) and triple quadrupole (QqQ) analyzers have been widely used to this aim. Given the high complexity of marine phenolics, MS^n^ spectrums help only partially to identify the polymerization degree and structure of phlorotannins. Coupling NMR and tandem mass spectrometry (MS^n^) with liquid chromatography is another strategy used to identify and characterize the chemical structure of this group of compounds. No less important are the advances in clean and efficient extraction methods, as well as the fractionation and purification strategies, which can promote the rational utilization of these compounds as bioactive components in functional foods, nutraceuticals and medicines. This is especially relevant since other compounds (carbohydrates, pigments, or toxic heavy metals) can be co-extracted with marine phenolics. Among the isolation techniques assayed, classical solid–liquid extraction using organic solvents is the most studied method. Alternatively, pressurized hot liquid extraction (PHLE) is a more recent option to obtain pure phlorotannins and bromophenols extracts, with lower environmental impact than solid-liquid extraction, but difficult to scale up to industrial production. 

Although deficiencies in polyphenol intake do not result in specific diseases, adequate consumption of polyphenols could confer health benefits, especially related to the prevention of non-communicable diseases. The reviewed studies have revealed the multi-targeted protective effect of marine phenolics against the most prevalent diseases, such as T2DM, obesity, metabolic syndrome, Alzheimer’s, or cancer, along with infectious diseases, among others. The modulatory activity of human gut microbiota has been also described, although few studies are currently available, and it would be desirable to expand them to address this aspect in depth.

Many studies have demonstrated the involvement of polyphenols in various multifactorial mechanisms underlying several diseases, due to their enzyme inhibitory effect along with their antidiabetic, antiobesity, antihypertensive, anti-inflammatory, anticancer, antimicrobial, or antiviral activities. This is an important difference compared to the available drugs used to treat most of the diseases, i.e., the ability of marine phenolics intervening in multiple pathways involved in the pathological processes. This reinforces their consideration in the pharmaceutical and cosmeceutical industries as drug substitutes. This step must be supported by the development of human studies since current understanding on the bioactivity of marine phenolics is almost exclusively based on the data available from the in vitro assays or cellular and animal models; hence, they cannot be extrapolated without reliable human clinical data.

The majority of the reported clinical trials aimed to ascertain the effect of marine phenolics on obesity and diabetes and there is not one on cancer or Alzheimer’s. Regarding the polyphenol types, phlorotannins bioactivity was much more explored than bromophenols; particularly eckols and their derivatives have shown to be promising. Therefore, it is essential to design clinical trials to confirm the current knowledge about the bioactivity of marine phenols, rule out adverse effects, and study their metabolism and bioavailability for their study is almost un-existent so far.

In conclusion, marine organisms represent an important polyphenol source with promising beneficial properties to ameliorate the prevalent non-communicable diseases such as diabetes, obesity, cancer, and neurodegenerative pathologies.

## Abbreviatures

A2780	Human ovarian carcinoma cell line
A549	Adenocarcinomic human alveolar basal epithelial cell line
Aβ	Amyloid-beta peptides
ABTS	2, 2′-Azino-bis (3-ethylbenzothiazoline-6-sulphonic acid)
ACC	Acetyl-CoA carboxylase
ACE	Angiotensin-I converting enzyme
AChE	Acetylcholinesterase
ACh	Acetylcholine
ACSL1	Adipose acyl-CoA synthetase 1
AD	Alzheimer’s disease
A-FABP	Adipocyte fatty acid binding protein
AGEs	Advanced glycation end-products
AIDS	Acquired immunedeficiency syndrome
Akt	Protein kinase B
AMPK	AMP-activated protein kinase
ASE	Ascorbic acid equivalents
ATF3	Transcription factor 3
B16F10	Murine melanoma cell line
BACE-1	Beta-site amyloid precursor protein cleaving enzyme 1
BCBM	β-Carotene bleaching
BChE	Butyrylcholinesterases
BDNF	Brain-derived neurotrophic factor
BT549	Human triple negative breast cancer cell line
C2C12	Mouse myoblast cell line
C8166	Human cancer cell line
CA	Chelating ability
CAA	Antioxidant assay for cellular antioxidant activity
CAT	Catalase
CEM-SS	Human lymphoblastic leukemia cell line
CLPAA	Cellular lipid peroxidation antioxidant activity assay
COSY	Homonuclear correlation spectroscopy
GC–MS	Gas chromatography–mass spectrometry
Glut4	Glucose transporter 4
C/EBPα	CCAAT/enhancer-binding protein alpha
COX-2	Cyclooxygenase-2
CPT1A	Carnitine palmitoyltransferase I
CVD	Cardiovascular disease
DNA	Deoxyribonucleic acid
DPPH	2,2-Diphenyl-1-picrylhydrazyl
E1-6	Human Jurkat clone cell line
EA.hy926	Human umbilical vein endothelial cell line
ERK	Extracellular signal-regulated kinase
FABP4	Fatty acid binding protein 4
FAS	Fatty acid synthase
FATP1	Fatty acid transport protein-1
FemX	Human malignant melanoma cell line
FFAs	Free fatty acids
FRAP	Ferric reducing antioxidant power
GAE	Gallic acid equivalents
Glut4	Glucose transporter 4
GPx	Glutathione peroxidase
GR	Glutathione reductase
GSK3β	Glycogen synthase kinase 3β
H157	Human oral squamous cell carcinoma cell line
H9	Human embryonic stem cell line
HCT-15	Human colon adenocarcinoma cell line
HCT-116	Human colon cancer cell line
HDL-C	HDL-cholesterol
HeLa	Human cervical cervix cancer cell line
HepG2	Human hepatocellular carcinoma cell line
HIV-1	Human immunodeficiency virus-1
HMGCoA	3-Hydroxyl-methyl glutaryl coenzyme A
HPLC–DAD–ESI/MS	High performance liquid chromatography–Diode array–Electrospray ionization–Mass spectrometry
HSQC	Heteronuclear single-quantum coherence spectroscopy
HT-22	Mouse hippocampal neuronal cell line
HT29	Human colon adenocarcinoma cell line
HUVEC	Human umbilical vein endothelia cells
IL	Interleukin
Ins-1	Rat insulinoma cell line
IR	Infrared spectroscopy
IRS1	Insulin receptor substrate 1
JNKs	c-Jun N-terminal kinases
K562	Human myelogenous leukemia cell line
L6	Rat skeletal myoblast cell
L929	Mouse fibroblasts cell line
LC–ESI–MS/MS	Liquid chromatography–Electrospray ionization–tandem Mass spectrometry
LDL-C	LDL-cholesterol
LMW	Low molecular weight
LoVo	Human colon cancer cell line derived from supraclavicular lymph node metastasis
LPIA	Lipid peroxidation inhibition assay
LS174	Human colon adenocarcinoma cell line
MAPK	Mitogen-activated protein kinase
MC3T3-E1	Mouse osteoblastic cell line
MetS	Metabolic syndrome
MMPs	Matrix metalloproteinase
MNNG	Human osteosarcoma cell line
NBT	Superoxide anion scavenging test
MCF-7	Human breast adenocarcinoma cell line
MDA-MB-231	Human breast adenocarcinoma cell line
MIC	Minimum inhibitory concentration
NFkB	Nuclear factor kappa B
NMR	Nuclear magnetic resonance
Neuro 2A	Mouse neuroblastoma cell line
NO	Nitric oxide
NOESY	Nuclear overhauser spectroscopy
iNOS	Inducible nitric oxide synthase
ORAC	Oxygen radical absorbance capacity
PANC-1	Human pancreatic carcinoma cell line
Panc89	Human pancreatic carcinoma cell line
PancTU1	Human pancreatic carcinoma cell line
PC12	Rat neuronal cell line
PC3	Human prostate cancer cell line
PCNA	Proliferating cell nuclear antigen
PGE2	Prostaglandin E2
PGU	Phloroglucinol units
PI3K	Phosphoinositide 3-kinase
PPARγ	Proliferator activated receptor gamma
PTP1B	Protein tyrosine phosphatase 1B
RAGE	Receptor for advanced glycation end-products
RAW 264.7	Murine macrophage cell line
RINm5F	Rat insulinoma cell line (pancreatic β-cells)
ROS	Reactive oxygen species
RP	Reducing power
RRLC-ESI-MS	Rapid resolution liquid chromatography coupled to mass spectrometry detection with negative ion electrospray ionization
RT	Reverse transcriptase
SaOS-2	Human osteosarcoma cell line
SKBR3	Human breast cancer cell line
SK-N-SH	Human neuroblastoma cell line
SKOV3	Human ovarian carcinoma cell line
SOD	Superoxide dismutase
SREBP1	Sterol regulatory element binding protein 1
SREBP-1c	Sterol regulatory element binding protein -1c
SRSA	Superoxide radical scavenging assay
STZ	Streptozotocin
SW480	Human colon cancer cell line
3T3-L1	Mouse adipocyte cell line
T2DM	Type 2 diabetes mellitus
TAA	Total antioxidant capacity
TBARs	Thiobarbituric acid reactive substances
TC	Total cholesterol
TEAC	Trolox equivalent antioxidant capacity
TG	Triglycerides
TLR	Toll-like receptor
TNFα	Tumor necrosis factor alpha
TOCSY	Total correlation spectroscopy
UCP-1	Uncoupling protein 1
UPLC	Ultra-performance liquid chromatography
UPLC–ESI–QTOF/MS	Ultra-performance liquid chromatography–Electrospray ionization–Quadrupole-time-of-flight high definition–Mass spectrometry
UPLC–MS	Ultra-performance liquid chromatography–Mass spectrometry
UPLC–MS/MS–TIC	Ultra-performance liquid chromatography–tandem Mass spectrometry–Total ion chromatogram
VEGF	Vascular endothelial growth factor
VERO	Green monkey kidney cell line
VSMC	Human vascular smooth muscle cell line
